# Comparative transcriptomics elucidates the cellular responses of an aeroterrestrial zygnematophyte to UV radiation

**DOI:** 10.1093/jxb/erae131

**Published:** 2024-03-23

**Authors:** Anna Busch, Jennifer V Gerbracht, Kevin Davies, Ute Hoecker, Sebastian Hess

**Affiliations:** Department of Biology, University of Cologne, Zülpicher Str. 47b, D-50674 Cologne, Germany; Department of Biology, University of Cologne, Zülpicher Str. 47b, D-50674 Cologne, Germany; The New Zealand Institute for Plant and Food Research Limited, Private Bag 11600, Palmerston North 4442, New Zealand; Institute for Plant Sciences and Cluster of Excellence on Plant Sciences (CEPLAS), Biocenter, University of Cologne, Zülpicher Strasse 47b, D-50674, Cologne, Germany; Department of Biology, University of Cologne, Zülpicher Str. 47b, D-50674 Cologne, Germany; University of Helsinki, Finland

**Keywords:** Lignin, peroxidase, phenolics, phenylpropanoid, streptophyte algae, UV radiation, UVR8, *Zygnematophyceae*

## Abstract

The zygnematophytes are the closest relatives of land plants and comprise several lineages that adapted to a life on land. Species of the genus *Serritaenia* form colorful, mucilaginous capsules, which surround the cells and block harmful solar radiation, one of the major terrestrial stressors. In eukaryotic algae, this ‘sunscreen mucilage’ represents a unique photoprotective strategy, whose induction and chemical background are unknown. We generated a *de novo* transcriptome of *Serritaenia testaceovaginata* and studied its gene regulation under moderate UV radiation (UVR) that triggers sunscreen mucilage under experimental conditions. UVR induced the repair of DNA and the photosynthetic apparatus as well as the synthesis of aromatic specialized metabolites. Specifically, we observed pronounced expressional changes in the production of aromatic amino acids, phenylpropanoid biosynthesis genes, potential cross-membrane transporters of phenolics, and extracellular, oxidative enzymes. Interestingly, the most up-regulated enzyme was a secreted class III peroxidase, whose embryophyte homologs are involved in apoplastic lignin formation. Overall, our findings reveal a conserved, plant-like UVR perception system (UVR8 and downstream factors) in zygnematophyte algae and point to a polyphenolic origin of the sunscreen pigment of *Serritaenia*, whose synthesis might be extracellular and oxidative, resembling that of plant lignins.

## Teaser

UV-induced gene expression points to a phenolic origin of extracellular sunscreen pigments in zygnematophyte algae, and highlights the importance of secreted peroxidases in the closest relatives of land plants.

## Introduction

The conjugating green algae (*Zygnematophyceae*) represent an algal class of ~4000 described species, which inhabit diverse freshwater-fed systems (Hall and McCourt, 2015). They colonize standing waters, from eutrophic lakes to dystrophic moorlands, but also thrive on terrestrial surfaces (e.g. rocks, bark, and deadwood) and even glaciers. Hence, these algae display a wide ecological variation, and, at the same time, relatively simple growth forms (unicells or filaments). It appears that their ecological variation is likely to be underpinned by physiological specialties that evolved in distinct zygnematophyte taxa.

Furthermore, the zygnematophytes are the key to understanding the evolution of plant metabolism and the process of terrestrialization, as these algae represent the sister clade of the land plants (Wodniok *et al.*, 2011; Timme *et al.*, 2012; Ruhfel *et al.*, 2014; Wickett *et al.*, 2014; Leebens-Mack *et al.*, 2019). Indeed, the zygnematophytes are gaining increasing attention by various biological disciplines. Well-studied aspects include cell wall synthesis and composition (Domozych, 2014; Domozych *et al.*, 2014), physiological reactions to abiotic stressors and metabolic networks (Pichrtová *et al.*, 2014; de Vries *et al*., 2020; de Vries and Ischebeck, 2020; Permann *et al.*, 2022), genome evolution (Cheng *et al.*, 2019), and phylogenetics (Gontcharov *et al.*, 2004; Gontcharov, 2008; Hess *et al.*, 2022). Overall, it is thought that algal (pre-)adaptations concerning various cellular and metabolic traits might have paved the way for the evolution of the land plants (de Vries and Archibald, 2018; Jiao *et al.*, 2020). However, so far, relatively few zygnematophyte species (e.g. from the genera *Mesotaenium*, *Mougeotia*, *Spirogloea*, *Penium*, and *Zygnema*) have been subjected to in-depth genomic or transcriptomic analyses (Cheng *et al.*, 2019; Jiao *et al.*, 2020; Fürst-Jansen *et al.*, 2021; Dadras *et al.*, 2023a; Feng *et al.*, 2023, Preprint). These revealed taxon-specific differences (e.g. the triploid genome of *Spirogloea*) and showed that our picture of the zygnematophyte specialized metabolism, perception of environmental factors, and signaling is still fragmentary. This is not surprising given the enormous diversity of zygnematophytes and their lifestyles. Hence, we need data of various species to tell apart common and species-specific traits, and to gain insights into how certain zygnematophyte lineages adapted to their specific environments.

Aeroterrestrial zygnematophytes are of particular interest as they cope with abiotic stressors that might have been crucial during the evolution of land plants, namely limited water supply, frequent desiccation, high temperature amplitudes, and intense sunlight. Interestingly, several distinct zygnematophyte lineages exhibit a terrestrial lifestyle or thrive in otherwise extreme habitats such as glaciers and alpine lakes (Remias *et al.*, 2012a; Aigner *et al.*, 2013; Garduño-Solórzano *et al.*, 2021; Busch and Hess, 2022). Under these conditions, high light exposure is a serious stressor as UV radiation (UVR) damages nucleic acids and proteins, and thus can disturb vital metabolic functions (Karentz *et al.*, 1991; Lao and Glazer, 1996; Buma *et al.*, 2003). It appears that some zygnematophyte lineages evolved photoprotective strategies that reduce cellular damage under high light conditions. For example, representatives of the distantly related genera *Ancylonema*, *Temnogametum*, and *Zygogonium* produce colorful intracellular compounds that are thought to be sunscreens (Newsome and van Breemen, 2012; Remias *et al.*, 2012b; Aigner *et al.*, 2013; Garduño-Solórzano *et al.*, 2021). It has been established that these compounds, identified as purpurogallin derivates or gallic acid polymers, have a phenolic origin and effectively absorb light and UVR. However, the biosynthesis of these pigments is still unknown, as zygnematophytes from extreme habitats are difficult to cultivate (Remias and Procházková, 2023) and no associated genomic and metabolomic data are available (but see Bowles *et al.*, 2023, Preprint).

**Fig. 1.**
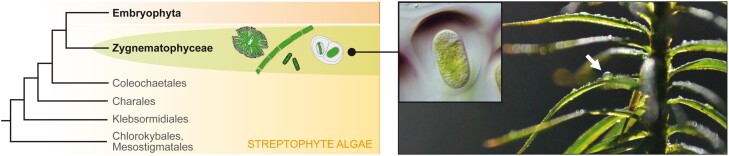
Phylogenetic affinity and aeroterrestrial lifestyle of *Serritaenia* species. *Serritaenia* belongs to the *Zygnematophyceae*, which have a key position in the streptophyte phylogeny. Several *Serritaenia* species colonize bryophytes (arrow) and form pigmented ‘sunscreen mucilage’ (inset). Topology of the phylogenetic tree according to Wickett *et al.* (2014).

A very different photoprotective strategy can be found in the genus *Serritaenia*, whose members inhabit forests, moorlands, and heathlands in temperate regions of Europe and North America (Busch and Hess, 2021). These unicellular zygnematophytes form gelatinous colonies that stick to plant and rock surfaces, and produce a colorful extracellular pigment (Fig. 1). The pigment is often secreted in a directional manner and, as shown by microspectrophotometry, effectively blocks UVR. So far, *Serritaenia* is the only known zygnematophyte lineage able to produce this extracellular ‘sunscreen mucilage’, and hence represents a unique organismal system. The closest analogy can be found in the photoprotective sheath pigments (gloeocapsin and scytonemin) of cyanobacteria (Proteau *et al.*, 1993; Storme *et al.*, 2015), which, however, have different properties and are unlikely to occur in eukaryotic algae. They can be readily extracted with methanol/ethyl acetate mixtures or acetone, while the sunscreen pigment of *Serritaenia* appeared to be resistant to various solvents and harsh acidic hydrolysis (Busch and Hess, 2021), and was intractable to standard chemical analyses. However, *Serritaenia* species can be cultivated in the laboratory and triggered by artificial UVR to produce their extracellular pigmentation. Thus, these algae are excellent laboratory models to study the reaction of zygnematophytes to UVR and to gain insights into the formation of the sunscreen mucilage by transcriptomic methods.

Here, we generated a *de novo* transcriptome of *Serritaenia testaceovaginata* and explored the transcriptional reactions of this species to moderate UVR exposure. Besides general cellular processes such as DNA repair, photosynthesis, and reactive oxygen species (ROS) scavenging, we examined the photoreceptor systems and the specialized metabolism of aromatic compounds. A special focus is set on highly regulated oxidative enzymes known from lignin formation in higher plants, whose functions in zygnematophytes are still unknown.

## Materials and methods

### Experimental set-up

Algae were pre-grown in tissue culture flasks in liquid culture medium KW (Supplementary Table S1) at 30 µmol m^–2^ s^–1^ photosynthetically active radiation (PAR) (LinearZ SunLike LEDs, 5700 K, Lumitronix, Hechingen) with a 14/10 h light/dark cycle for 4 weeks. Before the start of the light experiment, the cells were transferred to Petri dishes with fresh medium and acclimatized to higher PAR conditions (120 µmol m^–2^ s^–1^, 14/10 h) for 10 d. During the experiment, the algae were exposed to the above-mentioned LEDs and the UVB Broadband TL fluorescent tube lamp (20 W, Philips, Hamburg), resulting in 120 µmol m^–2^ s^–1^ PAR, 400 µW cm^–2^ UV-B (280–315 nm), and 150 µW cm^–2^ UV-A (315–400 nm). While PAR was applied in the regular 14/10 h photoperiod, UVR was applied for 4 h at noon. Control cells were covered with a Makrolon® polycarbonate plate that blocks UVR <390 nm (Busch and Hess, 2021). The experiment was run in triplicate for 3 d at 16 °C. After the third UVR exposure, when the algae displayed slight extracellular pigmentation, all samples were subjected to RNA isolation. Brightfield microscopy and photo-documentation of experimental cultures were carried out with the Motic AE2000 inverted microscope (Motic, Hong Kong) equipped with a MikroLive 6.4MP CMOS camera (MikroLive, Oppenau).

### RNA isolation and RNA sequencing

Algal cells were collected by centrifugation (500 *g*, 5 min), lysed as described in Gerbracht *et al.* (2022), and subjected to RNA isolation with the TRIzol Reagent (Thermo Fisher Scientific Inc., Waltham, MA, USA) according to the manufacturer’s protocol. The RNA samples (see Supplementary Fig. S1 for gel picture) were submitted to the Cologne Center for Genomics (Cologne, Germany) for paired-end mRNA library preparation (Illumina TruSeq mRNA stranded, Illumina, San Diego, CA, USA) and RNA sequencing (RNA-seq; ~20 million reads/sample) on a NovaSeq 6000 platform (Illumina).

### Transcriptome assembly

K-mer-based error correction was done with R-Corrector version 1.0.4 (Song and Florea, 2015), and quality and adapter trimming with Trim Galore version 0.6.6 (https://github.com/FelixKrueger/TrimGalore). Processed reads from all conditions were pooled (267 756 958 reads in total) and assembled *de novo* with Trinity version 2.0.6 (Grabherr *et al.*, 2011) in the strand-specific mode. To detect potential contaminants, the resulting transcriptome was blasted against the nt database (megablast, version 2.20.1), and sequences with a length >100 nucleotides and >95% identity with ribosomal, bacterial, or viral sequences were removed. ORFs were predicted with Transdecoder version 2.1.0 (https://github.com/TransDecoder/TransDecoder). Transcriptome assembly statistics were obtained with Trinity toolkit utilities (TrinityStats.pl) and BUSCO version 4.0.6 (Seppey *et al.*, 2019).

### Functional annotation

The predicted ORF sequences were compared with the nr database (release 2020_06) using DIAMOND blastp version 2.0.7 (Buchfink *et al.*, 2021) with an e-value cut-off of 1 × 10^–6^. Furthermore, we applied EggNOG mapper version 2.1.7 (Cantalapiedra *et al.*, 2021) for a gene ontology (GO) annotation in the DIAMOND mode. InterProScan version 5.22-61.0 (Blum *et al.*, 2021) was used running the following analyses: CDD-3.14, Coils-2.2.1, Gene3D-3.5.0, Hamap-201605.11, MobiDBLite-1.0, PANTHER-11.1, Pfam-30.0, PIRSF-3.01, PRINTS-42.0, ProDom-2006.1, ProSitePatterns-20.119, ProSiteProfiles-20.119, SFLD-2, SignalP_EUK-4.1, SMART-7.1, SUPERFAMILY-1.75, TIGRFAM-15.0, and TMHMM-2.0c. The knumbers were annotated using KAAS annotation (Moriya *et al.*, 2007) in SBH (single-directional best hit) mode using defined organisms as reference (organism abbreviations: ath, boe, gmx, rcu, pop, qsu, vvi, sly, psom, osa, zma, mus, ppp, cre, mng, apro, olu, mpp, cme, ccp, mdm, spen, nta, and to). Furthermore, Ghost Koala version 2.2 (Kanehisa *et al.*, 2016) was used with the KEGG (Kyoto Encyclopedia of Genes and Genomes) database ‘genus_prokaryotes+family_eukaryotes’ and Kofam Koala version 101.0 (Aramaki *et al.*, 2020) with an e-value cut-off of 0.01. To get the most complete picture (see UpSet plot in Supplementary Fig. S2), retrieved knumbers from KAAS, Ghost Koala, and Kofam Koala were merged and the resulting dataset was used for KEGG pathway mapping using *Arabidopsis thaliana* as a reference (Kanehisa and Sato, 2020; Kanehisa *et al.*, 2022). Transmembrane domains and signal peptides were predicted for selected protein sequences with DeepLoc 2.0 (Almagro Armenteros *et al.*, 2017; Thumuluri *et al.*, 2022) in high-quality mode, DeepTMHMM (Hallgren *et al.*, 2022, Preprint), and SignalP 6.0 (Teufel *et al.*, 2022). Binding sites and the active site of class III peroxidase were predicted by conserved domain search on NCBI (Lu *et al*., 2020). Furthermore, for the first 50 up-regulated genes, the predicted ORF sequences were compared with the refseq_protein database (15 January 2024) using blastp version 2.15.0 (Altschul *et al*., 1990) with an e-value cut-off of 1 × 10^–10^. The hit with the lowest e-value with functional information from eukaryotes was chosen, while annotations from plants and green algae were preferred.

### Homology searches of specific protein groups

Homologs of enzymes scavenging ROS and class III peroxidases were searched in the output files of EggNOG mapper and InterProScan by EC number and protein name searches. Homologs of photosynthesis proteins and enzymes of specialized metabolite pathways were searched by knumber annotation (see above) and KEGG pathway mapping (map00195, map00940, map00941, map00942, map00944, map00943, and map00965). Furthermore, photosynthesis-associated proteins (from *A. thaliana* and *Chlamydomonas reinhardtii*), jasmonate pathway-related proteins (from *A. thaliana* and *Glycine max*), photoreceptors and photoreceptor-associated proteins (from *A. thaliana*, *C. reinhardtii*, and *Mougeotia scalaris*), and proteins related to the biosynthesis of scytonemin and mycosporine-like amino acids (from cyanobacteria) were used to search for homologs in *S. testaceovaginata* by blastp searches (Altschul *et al.*, 1990). For blastp searches, only putative homologs with an e-value <1 × 10^–10^, a percentage identity >30%, and a minimal alignment length >50% of the query sequence were chosen.

### Differential expression analysis

The processed reads were mapped to the *de novo* transcriptome with bowtie2 version 2.4.1 (Langmead and Salzberg, 2012), and transcript abundance was quantified with Salmon version 1.14.1 in the alignment-based mode (Patro *et al.*, 2017). Transcript-level abundances, estimated counts, and transcript lengths were imported with tximport version 1.18.0 (Soneson *et al.*, 2016) and summarized into a matrix. Only contigs with a counts per million (CPM) >1 in two or more samples were kept. The differential expression analysis was carried out with DESeq2 version 1.30.0 (Love *et al.*, 2014).

### Global enrichment analyses

GO term enrichment analysis was performed with GOseq version 1.42.0 (Young *et al.*, 2010). The sequence lengths required for the analysis were computed with the script ‘fasta_seq_length.pl’ from the Trinity toolkit utilities. GO terms were retrieved by EggNOG mapper version 2.1.7 (Cantalapiedra *et al.*, 2021). The adjusted *P*-value was set to <0.01 and log2fold change (FC) >1 (up-regulated) or <1 (down-regulated). Up-regulated genes within the term ‘Response to UV’ were assigned to putative homologs based on EggNOG and InterProScan descriptions, and blastp searches against the UniProtKB/Swiss-Prot database (performed in May 2022). For blastp searches, only putative homologs with an e-value <1 × 10^–10^, a percentage identity >30%, and a minimal alignment length >50% of the query sequence were chosen.

### Protein structure predictions and phylogenetic analyses

Protein structure predictions were performed with I-TASSER (Yang and Zhang, 2015) and visualized with iCn3D (J. Wang *et al.*, 2020). For phylogenetic analysis of class III peroxidases, we created a multiple sequence alignment with streptophyte sequences (all algae and selected embryophytes) from RedOxiBase (Savelli *et al.*, 2019), published sequences from Morgenstern *et al.* (2008), two selected sequences from the RCSB protein data bank (3HDL, 1BGP), and homologs from the algal transcriptomes and genomes listed in Supplementary Table S2. The latter homologs were extracted by Blast searches (e-value <1 × 10^–10^, length >250 amino acids, percentage identity >30%) with the sequence of StesPRX01 (TRINITY_DN14219) as query. The sequences were aligned with MAFFT version 7.471 (Katoh and Standley, 2013) in ‘auto’ mode and trimmed with trimAl version 1.4.rev15 (Capella-Gutiérrez *et al.*, 2009) using the ‘automated1’ setting. The substitution model with the best fit was determined by the ModelFinder function of IQ-TREE version 4.5.1 (Minh *et al.*, 2020), and maximum likelihood phylogenies were inferred with IQ-TREE. After manually reducing sequence redundancy, a final phylogenetic analysis (124 sequences, 248 sites; alignment in Supplementary Dataset S1) was run with the substitution model Q.pfam+R7 and 1000 bootstrap replicates.

Supporting phylogenetic analyses for shikimate and betalain pathway-related genes were conducted within the Geneious Prime sequence analysis software package (Biomatters, New Zealand). The candidate transcriptome sequences were hand-annotated and then translated to provide the deduced amino acid sequences, which were aligned using ClustalOmega or MUSCLE, and the alignments manually adjusted as necessary. Phylogenetic trees were inferred from conserved regions using MrBayes (Ronquist *et al.*, 2012) with an outlier sequence and the default parameters. For assisting with assigning putative function, the trees contained sequences of confirmed function from land plants, along with related sequences from other species of interest. Sequence accession numbers are given within the phylogenies.

## Results and discussion

### Capturing the reaction of *Serritaenia* to UV radiation by RNA-seq

**Fig. 2.**
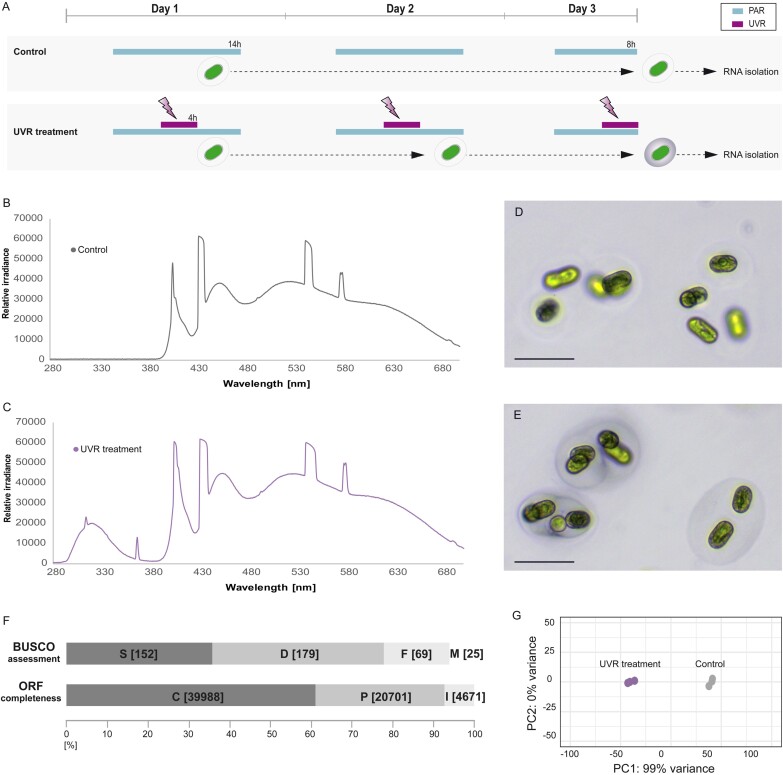
Experimental set-up and *de novo* transcriptome assembly of *Serritaenia testaceovaginata*. (A) PAR/UVR exposure of cells under the two experimental conditions over the course of 3 d. (B) Relative spectral power distribution under the PAR-only treatment (‘Control’). (C) Relative spectral power distribution under the PAR+UVR treatment (‘UVR treatment’). (D and E) Cells of *S. testaceovaginata* from the control (D) and the UVR treatment (E) at harvest. Scale bars 50 µm. (F) BUSCO assessment of the assembled transcriptome (top) and completeness of the predicted ORFs (bottom). The BUSCO analysis was performed with the ‘Viridiplantae’ dataset. The absolute numbers of single-copy orthologs for the categories S (complete and single-copy), D (complete and duplicated), F (fragmented), and M (missing) are shown in brackets. The absolute numbers of ORFs (bottom) for the categories C (complete), P (partial), and I (internal) are also shown in brackets. (G) Principal component analysis (PCA) based on the expression level of all transcripts for each replicate included in the experiment.

Based on previous observations on the visible reactions of *S. testaceovaginata* to UVR (Busch and Hess, 2021), we treated the algae with two well-defined conditions over 3 d (Fig. 2A). Control cells experienced a daily 14 h photoperiod of PAR, with a continuous spectrum provided by sun-mimicking LEDs (Fig. 2B). The UVR treatment was characterized by the same PAR spectrum but supplemented with UVR for 4 h per photoperiod (Fig. 2A). The applied UVR had its main emission in the UV-B and far UV-A region, plus a narrow secondary peak at 365 nm (Fig. 2C). While the mucilage of control cells remained colorless (Fig. 2D), cells under the UVR treatment displayed a faint bluish pigmentation after the third photoperiod of the experiment (Fig. 2E). This indicated the ongoing formation of *Serritaenia*’s typical photoprotection triggered by UVR (Busch and Hess, 2021). At this stage, *Serritaenia* cells from both conditions were processed for Illumina RNA sequencing. As there was no reference genome of *Serritaenia* available, we assembled a *de novo* transcriptome from read data of both conditions (six samples). The transcriptome was 77.9% complete according to the BUSCO analysis (Benchmarking Universal Single-Copy Orthologs; Seppey *et al.*, 2019) with the *Viridiplantae* dataset as reference (Fig. 2F). We also checked the completeness of the >60 000 predicted ORFs and found that >60% of them were annotated as complete protein sequences (Fig. 2F). The number of predicted ORFs might seem high, but roughly aligns with the inferred gene numbers of other zygnematophytes (this varies strongly across species from ~11 000 to >50 000 genes; Feng *et al*., 2023, Preprint). However, it has to be noted that the ORFs of a *de novo* assembled transcriptome cannot be equated to the gene content of the organism. Yet, our *de novo* transcriptome of *S. testaceovaginata* should provide a fairly complete picture of the relevant transcriptomic landscape in this species, and served well for the differential expression analysis. As shown by a principal component analysis, the replicates of the two experimental conditions grouped in two tight and distinct clusters (Fig. 2G), indicating that the experimental set-up led to consistent reactions of the algae under the chosen conditions.

### UV radiation triggers cellular reprogramming, repair, and unexpected signaling components

**Fig. 3.**
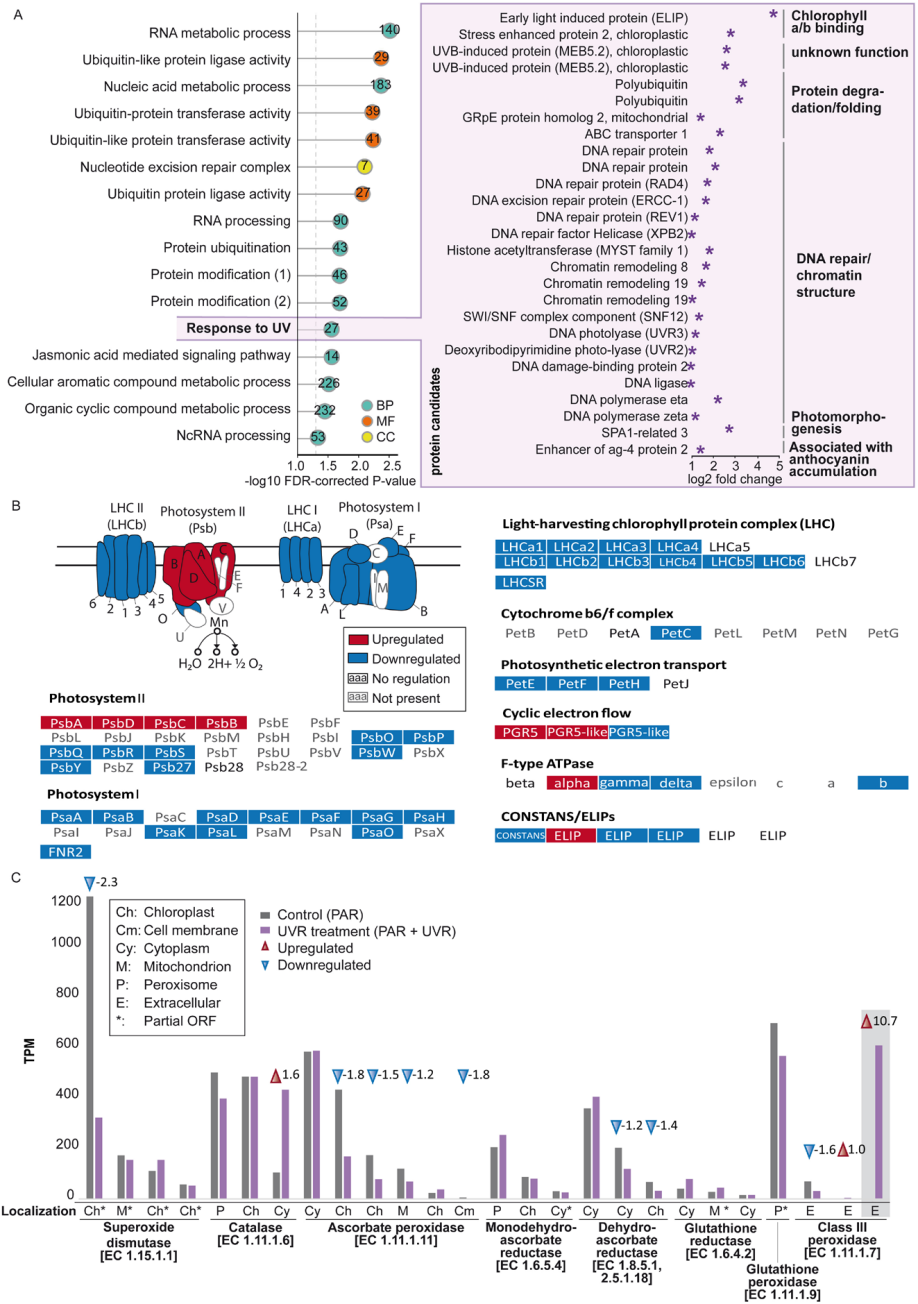
Gene ontology (GO) enrichment analysis, and gene regulation of the photosynthetic machinery and anti-ROS factors. (A) Enriched GO terms associated with up-regulated genes under the UVR treatment. GO terms are ranked according to the –log10 false discovery rate (FDR)-corrected *P*-value. Only terms with an FDR-corrected *P*-value <0.05 are shown. The numbers in the circles indicate the number of contigs of up-regulated genes associated with the listed GO terms. The color of the circles indicates the ontology of the respective term: turquoise (biological process, BP), yellow (cellular component, CC), orange (molecular function, MF). Functionally annotated genes of the GO term ‘Response to UV’ are shown in detail with their up-regulation under the UVR treatment (log2FC depicted by violet stars). (B) Regulation of the photosynthetic machinery under the UVR treatment versus the control illustrated by color-coded proteins/boxes: red (up-regulated, log2FC ≥1, adjusted *P*-value <0.001), blue (down-regulated, log2FC ≤1, adjusted *P*-value <0.001), white with black letters (no significant regulation, log2FC –1 to 1, adjusted *P*-value <0.001). Proteins with gray letters could not be recovered in the transcriptome. (C) Detected anti-ROS factors with their expression levels under the two conditions in transcripts per million (TPM) and predicted cellular localization. Red and blue arrowheads indicate up-regulation (log2FC ≥1, adjusted *P*-value <0.001) and down-regulation (log2FC ≤1, adjusted *P*-value <0.001), respectively, with the log2FC of the respective gene. Cellular localizations predicted on the basis of partial ORFs are indicated by asterisks. The gray box highlights the class III peroxidase StesPrx01, which might have other functions (biosynthesis of phenylpropanoids; see main text).

We identified GO terms that were significantly enriched in up-regulated genes under the UVR treatment. The majority of these enriched GO terms were related to RNA processing, protein degradation (ubiquitination), and protein modification (Fig. 3A, left). This indicates major expressional changes and a pronounced reprogramming of the cells under UVR. The enriched GO term ‘Response to UV’ contained 27 up-regulated genes and was of particular interest as it allows for some comparisons with other well-studied systems (Fig. 3A, right; Supplementary Table S3). Several genes with a known function in DNA repair and chromatin remodeling were up-regulated, indicating that the applied UVR caused damage to the DNA of *Serritaenia* during this early phase of sunscreen production. In particular, this included factors of the eukaryote nucleotide excision repair pathway (Supplementary Fig. S3), namely DDB2, XPC, XPB, TFIIH2, XPF, and ERCC1 (global genome repair, GGR); and CSB, UVSSA, POLR2, XPG, and RPA (transcription-coupled repair, TCR). This pathway is responsible for the removal of various types of DNA damage caused by UVR exposure and other damaging agents (Kimura and Sakaguchi, 2006). Most genes of the eukaryote base excision repair and mismatch repair pathways, which are known to remove damaged bases and erroneous base mutations that arise during DNA replication and recombination (Kimura and Sakaguchi, 2006), did not show significant regulation (Supplementary Figs S4, S5). There were also pronounced reactions related to protein degradation and folding, especially factors with chaperone-like functions. This includes homologs of the mitochondrial GrpE2 protein and ABC transporter 1 (Cardazzo *et al.*, 1998), both of which were shown to be triggered by UV-B in plants and algae such as *A. thaliana* (Hu *et al.*, 2012) and *Volvox carteri* (Razeghi and Kianianmomeni, 2019), respectively. A specific search for differentially expressed heat shock protein (HSP) sequences in the *Serritaenia* transcriptome revealed 11 up-regulated HSP genes (Supplementary Table S4), all predicted to act as molecular chaperones assisting in a wide range of folding processes of proteins (Mayer and Bukau, 2005; Qiu *et al.*, 2006; Guo *et al.*, 2020). Beneficial effects of HSPs under UV-B stress were reported for plants and algae, such as *A. thaliana* (Swindell *et al.*, 2007), the marine diatom *Odontella sinensis* (Döhler *et al.*, 1995), and the cyanobacterium *Synechocystis* sp. (Balogi *et al.*, 2008). Furthermore, chloroplastic factors associated with UVR responses in higher plants were up-regulated in *Serritaenia* (Fig. 3A, right). Two Chl *a*/*b*-binding proteins (ELIP, early light-induced protein; and SEP2, stress enhanced protein 2), for example, accumulate upon UV-B exposure in vascular plants and prevent excess accumulation of free chlorophyll, thereby protecting against photo-oxidative damage (Heddad and Adamska, 2000; Hutin *et al.*, 2003; Sävenstrand *et al.*, 2004). These factors have also been found in chlorophyte green algae such as *V. carteri* (Razeghi and Kianianmomeni, 2019) and *C. reinhardtii* (Allorent and Petroutsos, 2017), and might represent a universal mechanism in the green chloroplasts of the *Viridiplantae* at least. Similar to these other organisms, *Serritaenia* has several ELIP genes, most of which, however, were not up-regulated. All in all, these results reveal that the zygnematophyte *Serritaenia* has a UVR-responsive toolkit dedicated to repair and protection that in many ways reflects that of higher plants. The GO terms enriched in the down-regulated genes included those for signaling, chloroplast restructuring, phosphor-related processes, sugar metabolism, and cell cycle-related processes (Supplementary Fig. S6), suggesting that *Serritaenia* under UVR stress slows down some energy-costly processes such as cell growth and multiplication.

A surprising finding was up-regulated components of the GO term ‘Jasmonic acid mediated signaling pathway’. In vascular plants, the hormone jasmonic acid (JA) functions in defense, growth, and stress response, and in *A. thaliana* JA levels were shown to increase upon UV-B exposure (Mackerness *et al.*, 1999). So far, JA has not been reported to be involved in UVR- or stress-related signaling cascades in streptophyte green algae. In fact, recent studies suggest that JA signaling evolved in land plants and is absent in algal relatives (Rieseberg *et al.*, 2022). Overall, the pattern of detected proteins involved in JA synthesis, transport, and perception is patchy in streptophyte algae (Holzinger and Becker, 2015; S. Wang *et al*., 2020). In *Serritaenia*, we found homologs of digalactosyldiacylglycerol synthase 1 (DGD1; chloroplast), OPDA reductases (OPR2 and OPR3, chloroplast), ‘Novel Interactor of JAZ’ (NINJA; nucleus), and NAC transcription factors (NAC019, NAC055, and NAC072; nuclear) to be up-regulated. However, given the lack of other important JA-related factors (Supplementary Tables S5, S6), the functions of these proteins in *Serritaenia* and other zygnematophytes remain elusive.

### Responses of the photosynthetic machinery and anti-ROS factors

We also analyzed the regulation of genes associated with the photosynthetic machinery in *Serritaenia* in response to UVR (Fig. 3B; Supplementary Table S7). Almost all components of PSI and PSII, light-harvesting complexes, the cytochrome *b*_6_*f* complex, and photosynthetic electron transport were down-regulated or not differentially expressed. A marked exception were the four genes encoding PsbA (D1), PsbD (D2), PsbC (cp43), and PsbB (cp47) in the reaction center of PSII, which were up-regulated under the UVR treatment. It is known from plants that UV-B radiation or strong PAR exposure leads to the inactivation of PSII, by damaging first the oxygen-evolving complex followed by the reaction center (Ohnishi *et al.*, 2005). The damaged proteins are then replaced by *de novo* synthesis (Hakala *et al.*, 2005). This appears to be a quite common effect in photosynthetic organisms, as even cyanobacteria lose D1 and D2 after the exposure to moderate levels of UV-B (Wu *et al*., 2011). Hence, the up-regulation of D1, D2, cp43, and cp47 points to a specific degradation of the PSII reaction center of *Serritaenia*, which is compensated by the *de novo* synthesis of the damaged components. By contrast, PSI seems not to be affected by UVR in that way, which is in accordance with observations on plants (Iwanzik *et al.*, 1983; Kulandaivelu and Noorudeen, 1983). However, two important components of the cyclic electron flow at PSI, PGR5 and PGR5-like, were up-regulated. As in higher plants, the cyclic electron transport in *Serritaenia* might eliminate excess electrons (potentially accumulating due to the damage on PSII), and thereby reduce chlorophyll excitation and oxidative damage. Taken together, under the UVR treatment, the photosynthetic machinery of *Serritaenia* seems to be specifically damaged by UV-B at the reaction center of PSII, which might be compensated by the replacement of broken proteins and cyclic electron flow. Other factors known to be involved in photoprotective quenching in algae and land plants, for example CONSTANS, LHCSR, and PSBS (Suárez-López *et al.*, 2001; Serrano-Bueno *et al.*, 2009; Alboresi *et al.*, 2010; Furukawa *et al.*, 2019; Tokutsu *et al.*, 2019), were not up-regulated, indicating that the chloroplast experiences specific damage rather than typical light stress.

Elevated PAR and harmful wavebands such as UV-B are known to initiate the formation of ROS within the cell. ROS can play a regulatory role in gene expression as a response to UV-B radiation (Green and Fluhr, 1995; Surplus *et al.*, 1998; Mackerness *et al.*, 1999), but also cause cell damage by the degradation of various biomolecules (Czarnocka and Karpiński, 2018). To prevent high concentrations of ROS, organisms contain a plethora of enzymes from different families which eliminate the different forms of reactive oxygen. In the *Serritaenia* transcriptome, we found homologs of all of these typical ROS scavengers and predicted their cellular localization (Fig. 3C; Supplementary Table S8). Most of the 27 candidates were down-regulated or not differentially expressed, including factors such as ascorbate peroxidases (APXs), glutathione reductases, and a glutathione peroxidase, which in *A. thaliana* were found up-regulated under UVR stress (Ulm *et al.*, 2004). It appears that glutathione-associated ROS scavenging does not play a marked role in *Serritaenia* under the UVR treatment. However, the down-regulation of superoxide dismutases (SODs), dehydroascorbate reductases (DHARs), and APXs in the algae reflects the situation of UVR-treated *A. thaliana* (Ulm *et al.*, 2004). Only two putative ROS scavengers were highly expressed and up-regulated under the UVR treatment, namely a cytoplasmic catalase and an extracellular class III peroxidase (StesPrx01) (Fig. 3C, StesPrx01 highlighted by a gray box). While catalases have a clear function as ROS-scavenging enzymes catalyzing the dismutation of hydrogen peroxide (H_2_O_2_) into water (H_2_O) and oxygen (O_2_), class III peroxidases can have various biological roles, including the biosynthesis of polyphenols such as lignin in the apoplast. Although lignin is unlikely to occur in zygnematophytes, class III peroxidases might have biosynthetic functions in *Serritaenia* as discussed below. Both the specific reaction of the photosynthetic machinery and the limited up-regulation of typical ROS scavengers indicate that *Serritaenia* under UVR treatment was not in a stage of broad physiological stress.

### How does *Serritaenia* sense light and UV radiation?

**Fig. 4.**
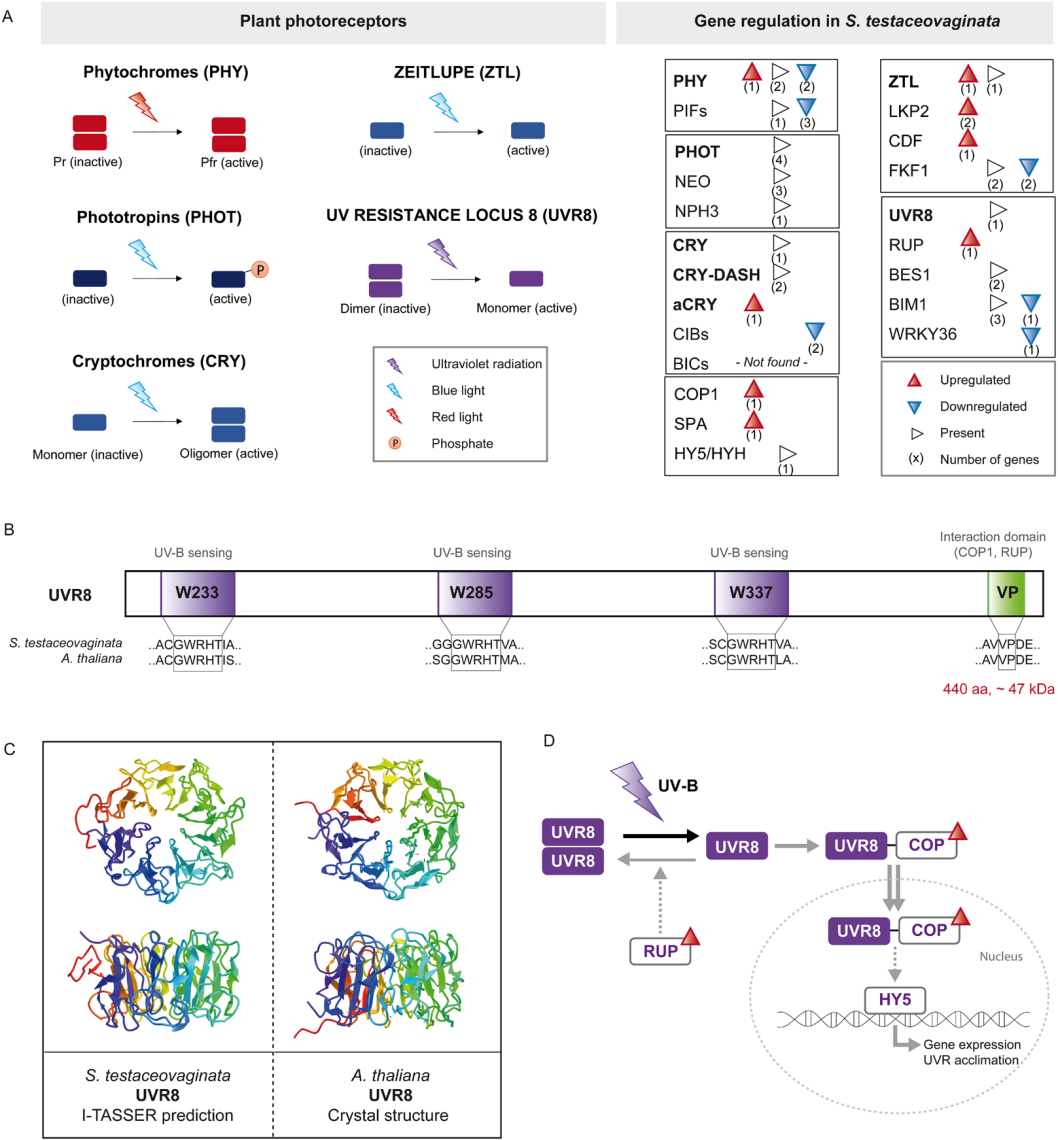
Presence and regulation of putative photoreceptor systems in *S. testaceovaginata*, with details on UVR8. (A) Schematic illustration of detected photoreceptors (left) and gene regulation of these photoreceptor systems including associated proteins (right). Regulation under the UVR treatment versus the control is indicated by colored arrowheads: red (up-regulated, log2FC ≥1, adjusted *P*-value <0.001), blue (down-regulated, log2FC ≤1, adjusted *P*-value <0.001), white (no significant regulation, log2FC –1 to 1, adjusted *P*-value <0.001). Numbers in parentheses below the arrowheads indicate the number of annotated ORFs (blastp, e-value cut-off of 1 × 10^–10^). PHY, phytochromes; PIFs, PHYTOCHROME INTERACTING FACTORS; PHOT, phototropins; NEO, neochromes; NPH3, NONPHOTOTROPIC HYPOCOTYL 3; CRY, cryptochromes; CRY-DASH, *Drosophila*, Arabidopsis, *Synechocystis*, human (DASH)-type cryptochromes; aCRY, animal-like cryptochromes; CIBs, CRYPTOCHROME2-INTERACTING-BASIC-HELIX–LOOP–HELIX proteins; BICs, blue-light inhibitors of cryptochromes; COP1, CONSTITUTIVE PHOTOMORPHOGENIC 1; SPA, SUPPRESSOR OF PHYA-105; HY5, ELONGATED HYPOCOTYL 5; HYH, HY5-HOMOLOG; ZTL, ZEITLUPE; LKP2, LOV KELCH PROTEIN 2; CDF, CYCLIC DOF FACTORS; FKF1, FLAVIN-BINDING, KELCH REPEAT, F-BOX 1; UVR8, UV-RESISTANCE LOCUS 8; RUP, REPRESSOR OF UV-B PHOTOMORPHOGENESIS; BES1, BRI1(BRASSINOSTEROID INSENSITIVE 1)-EMS (EXTRA MICROSPOROCYTES)-SUPRESSOR 1; BIM1, BES1-INTERACTING MYC-LIKE 1; WRKY36, WRKY DNA-BINDING PROTEIN 36. (B) Schematic diagram of the UVR8 photoreceptor from *S. testaceovaginata* with the three conserved tryptophan motifs (W233, W285, and W337) responsible for UV-B sensing and the VP domain responsible for the interaction with COP and RUP. (C) *In silico* structure prediction of the UVR8 photoreceptor from *S. testaceovaginata* next to the resolved crystal structure of the UVR8 from *A. thaliana* (pdb entry 4DNU) (Wu *et al.*, 2012). (D) Schematic diagram of the UVR8 signaling cascade triggered by UV-B radiation. Components up-regulated under the UVR treatment are marked by red arrowheads.

We identified components of all major plant photoreceptor systems in *Serritaenia*, including the red-light phytochrome (PHY) system, the blue-light phototropin (PHOT), cryptochrome (CRY), and ZEITLUPE (ZTL) systems, and the UV RESISTANCE LOCUS 8 (UVR8) system (Fig. 4A; Supplementary Table S9). With the exception of the phototropins, components of all photoreceptor systems showed expressional changes upon UV-B treatment (Fig. 4A, right). The interpretation of their functions in microalgae remains difficult, as several molecular components have mainly been studied in higher plants and are associated with processes such as germination and flowering (Kevei *et al.*, 2006; Paik and Huq, 2019). However, we know that the photoprotective reaction in *Serritaenia*, namely the synthesis of its extracellular sunscreen pigment, can be specifically triggered by UV-B radiation (Busch and Hess, 2021). Our *de novo* transcriptome of *Serritaenia* contained homologs of all components of the UVR8 photoreceptor system (Fig. 4A), which is known to perceive UV-B in land plants (Rizzini *et al.*, 2011) and might have a similar function in the distantly related chlorophyte algae (Tilbrook *et al*., 2016). The UVR8 homolog of *Serritaenia* showed a 65% sequence identity with the UVR8 receptor of *A. thaliana*, and its amino acid sequence contained all sequence motifs necessary for UV-B absorption (tryptophan residues at W233, W285, and W337) and interaction with regulatory factors [a valine–proline (VP) domain at the C-terminus] (Lau *et al.*, 2019) (Fig. 4B). Furthermore, the *in silico* predicted tertiary structure of the UVR8 homolog of *Serritaenia* is similar to the crystal structure of the UVR8 of *A. thaliana* (Wu *et al.*, 2012) (Fig. 4C), and its closest hit in the RCSB protein data bank (TM score 0.976) was the cryo-EM structure of the UV-B-activated UVR8 in complex with CONSTITUTIVE PHOTOMORPHOGENIC 1 (COP1) from *A. thaliana* (entry 7VGG). While the putative UVR8 receptor of *Serritaenia* did not show significant expressional changes under the UVR treatment, two components of the UVR8 signaling network, COP1 and REPRESSOR OF UV-B PHOTOMORPHOGENESIS (RUP), were clearly up-regulated (Fig. 4D). In plants, UVR8 dimers split into monomers upon UV-B exposure, which then interact with the E3 ubiquitin ligase COP1. This reduces the ubiquitin-mediated breakdown of the transcription factor ELONGATED HYPOCOTYL 5 (HY5) by COP1, and thus mediates the transcription of UV-B-responsive genes responsible for UV-B acclimation (Oravecz *et al.*, 2006; Tilbrook *et al.*, 2013; Liang *et al.*, 2019). RUP, in contrast, maintains the photoequilibrium when the UVR8 dimer/monomer cycling rate increases by facilitating the re-dimerization of UVR8, and thereby reduces again the transcription of UV-B-responsive genes (Liao *et al.*, 2020). This negative feedback loop can be understood as a counterbalancing reaction to UV-B-induced signaling. The existence of a UVR8 homolog with conserved functional sites and the pronounced regulation of COP1 and RUP as part of the UVR8 signaling network suggest that the zygnematophyte *Serritaenia* has a functional UVR8 system that may be a central component of its cellular reaction to harmful wavebands.

### Specialized metabolite pathways and their reaction to UV radiation

One of the most interesting questions is the metabolic origin of the extracellular sunscreen pigment of *Serritaenia*. A similar but probably analogous phenomenon can be found in the world of prokaryotes. Cyanobacteria produce sheath pigments such as scytonemin and gloeocapsin, which are formed and deposited in the extracellular matrix (Proteau *et al.*, 1993; Storme *et al*., 2015). The biosynthesis of the well-studied scytonemin is based on the cyanobacterial ‘Scytonemin gene cluster’ (Soule *et al*., 2007, 2009; Bennett and Soule, 2022). In *Serritaenia*, we did not detect most of these scytonemin-related genes, except potential homologs of trpA-E, aroB, and aroG, and a tyrosinase (Supplementary Table S10). Only one of the latter (trpE) was up-regulated under the UVR treatment. As expected due to the vast evolutionary distance of streptophyte green algae and cyanobacteria, and their fundamental differences in cellular organization, it seems unlikely that the sunscreen compound of *Serritaenia* is related to scytonemin biosynthesis. Another well-known group of sun screening compounds of algae are the colorless mycosporines and mycosporine-like amino acids (MAAs). They have been found in phylogenetically diverse phototrophs, including cyanobacteria, green algae, rhodophytes, dinoflagellates, and diatoms (Garcia-Pichel and Castenholz, 1993; Karsten *et al.*, 1998; Řezanka *et al.*, 2004; Hotter *et al*., 2018; Hartmann *et al.,* 2020). However, so far, there is no evidence of these compounds in zygnematophytes. We screened the *Serritaenia* transcriptome for MAA biosynthesis genes known from cyanobacteria (Balskus and Walsh, 2010; Singh *et al*., 2020), and found potential homologs of Ava3855, Ava3857, Ava3859, and NpR5599. Except Ava3859, these genes were not up-regulated under UVR treatment (Supplementary Table S11), and other relevant factors of MAA biosynthesis (Ava3856, Ava3858, NpR5597, and NpR5598) were not detected at all. It might well be that the *Zygnematophyceae* do not possess a functional MAA biosynthesis pathway.

**Fig. 5.**
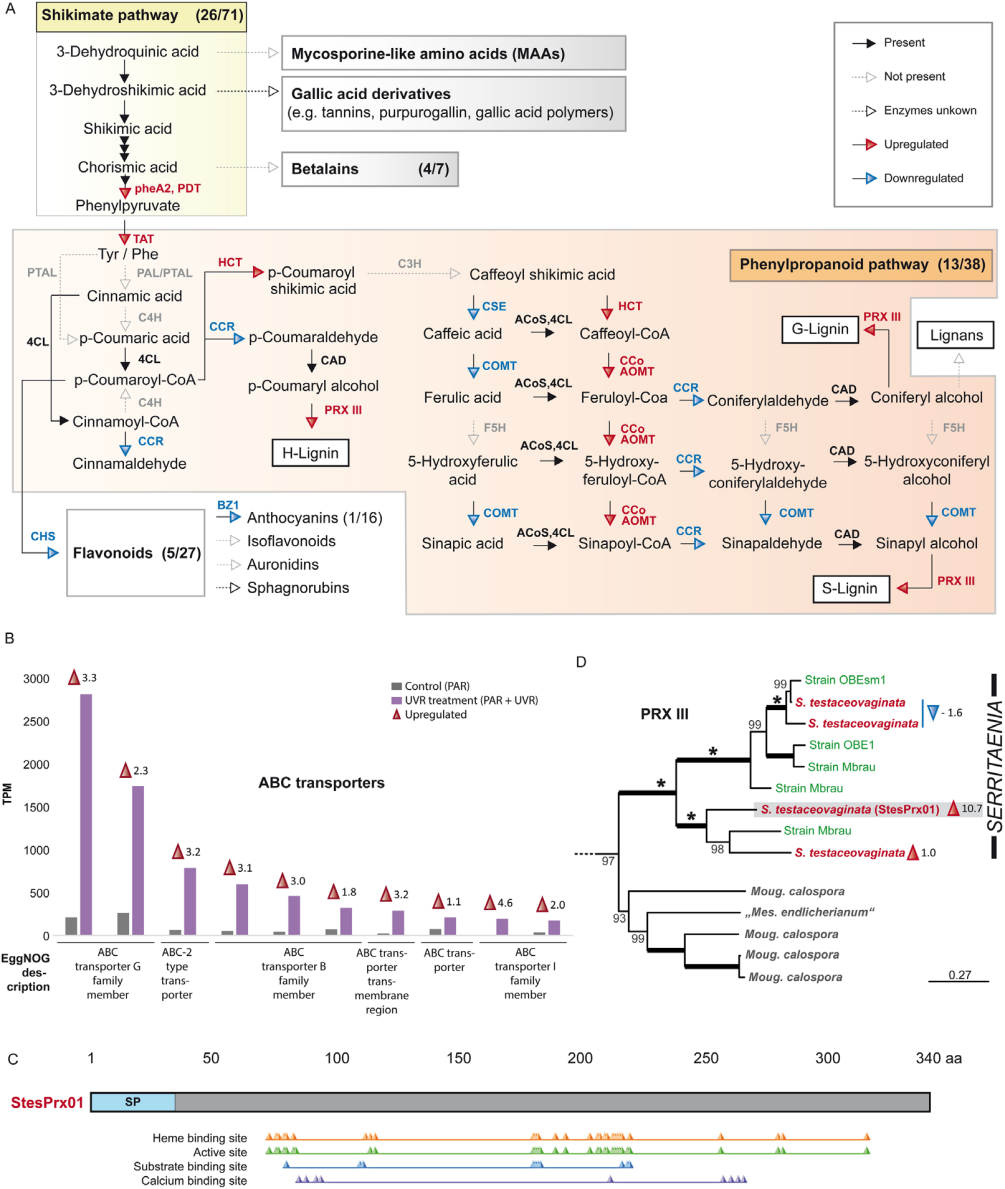
Regulation of specialized metabolite pathways and details on ABC transporters and class III peroxidases. (A) Schematic diagram of the shikimate pathway and downstream specialized metabolite pathways. Presence, absence, and regulation under the UVR treatment are indicated by the design and color of arrows (see key). The numbers in parentheses indicate the number of detected versus total knumbers of the respective pathway as a measure of completeness. PAL, phenylalanine ammonia-lyase; PTAL, phenylalanine/tyrosine ammonia-lyase; C4H, cinnamate 4-hydroxylase; 4CL, 4-coumarate-CoA ligase; CCR, cinnamoyl-CoA reductase; C3H, 4-coumarate 3-hydroxylase; CAD, cinnamyl-alcohol dehydrogenase; CSE, caffeoylshikimate esterase-like; COMT, catechol-*O*-methyltransferase; F5H, ferulate-5-hydroxylase; HCT, hydroxycinnamoyl CoA:shikimate/quinate hydroxycinnamoyltransferase; CCoAOMT, caffeoyl-CoA *O*-methyltransferase; ACoS, acyl-CoA synthetase; PRX III, class III peroxidase. Scale bars: 10 µm. (B) Top 10 most highly expressed ABC transporters under the UVR treatment with their expression levels in transcripts per million (TPM). Red and blue arrowheads indicate up-regulation (log2FC ≥1, adjusted *P*-value <0.001) and down-regulation (log2FC ≤1, adjusted *P*-value <0.001), respectively, with the log2FC of the respective gene. (C) Schematic diagram of the highly up-regulated class III peroxidase StesPrx01 (log2FC 10.7) depicting the signal peptide (SP), heme-binding site, active site, substrate-binding site, and calcium-binding site. (D) Section of the phylogenetic tree of class III peroxidases with genes of the genera *Serritaenia*, *Mougeotiopsis*, and *Mesotaenium* (the full tree is given in Supplementary Fig. S16). Ultrafast bootstrap values are shown at the branches, except when 100% (bold branches). Asterisks highlight potential gene duplication events in the genus *Serritaenia*, and colored arrowheads indicate up-regulation (log2FC ≥1) and down-regulation (log2FC ≤1) under the UVR treatment with the log2FC.

In the world of higher plants, most UVR-screening compounds have an aromatic origin and are derived from the shikimate pathway (Ferreyra *et al.*, 2021; Davies *et al.*, 2022). Zygnematophytes have also been shown to contain phenolic compounds, some of which are enriched under enhanced UVR and/or PAR levels (Aigner *et al.*, 2013; Pichrtová *et al.*, 2013; Holzinger *et al.*, 2018). However, the biosynthesis of such zygnematophycean compounds, including the colorful vacuolar gallic acid derivates (Remias *et al*., 2012b; Newsome and van Breemen, 2012), remains unknown. Interestingly, the GO term ‘Cellular aromatic compound metabolic process’ was enriched in *Serritaenia* under UV-B treatment (Fig. 3A). Hence, we studied the presence and regulation of candidate sequences for enzymes from diverse plant specialized metabolite pathways in *Serritaenia* upon UV-B radiation, with a focus on aromatic compounds. The shikimate pathway was fully recovered and the subsequent synthesis of aromatic amino acids (phenylalanine and tyrosine) from chorismic acid was strongly up-regulated (Fig. 5A; Supplementary Table S12), indicating an enhanced production of specialized metabolites derived from these aromatic amino acids. Aromatic amino acids are the primary building blocks for the phenylpropanoid pathway, which also leads to flavonoids such as anthocyanins, isoflavonoids, sphagnorubins, and auronidins, and to lignins (Vanholme *et al.*, 2019; Davies *et al.*, 2022). The colorful sphagnorubins and auronidins, in particular, are known from non-vascular plants, namely mosses (sphagnorubins) and liverworts (auronidins), and typically accumulate in the cell wall (Rudolph and Vowinkel, 1969; Rudolph *et al.*, 1981; Berland *et al.*, 2019). However, there were few components of the canonical plant polyphenolic metabolite pathways leading to flavonoids and anthocyanins in the transcriptome, and these pathways are probably not functional in *Serritaenia* (Fig. 5A). The few genes putatively assigned to these pathways were all down-regulated and may, in fact, also not be flavonoid related. In our protein phylogenies, the putative CHS homologs branch clearly outside the polyketide synthases from land plants that are known to be involved in phenylpropanoid biosynthesis (e.g. chalcone synthase, bibenzyl synthase, and stilbene synthase). Instead, one of the two candidate genes is nested in oxoalkylresorcinol synthases of bryophytes (Supplementary Fig. S7). The other one, which was strongly up-regulated during UVR treatment, did not branch with anything known. The *Serritaenia* gene annotated as CHI branches in the clade of fatty acid-binding protein b (FAPb), and not with the flavonoid enzyme clades of CHI and CHI-Like (Supplementary Fig. S8). Overall, the candidates annotated using KEGG as CHS, CHI, and UDP-glycosyltransferases (UGT) are not closely related to known genes with defined functions in flavonoid synthesis (Fig. 5A; Supplementary Figs S7–S9). Nevertheless, multiple flavonoid compounds were detected in the model zygnematophyte *Penium margaritaceum* and, as already suggested (Jiao *et al.*, 2020), this could be based on cryptic activities of known enzymes, novel enzymes, or even alternative biosynthetic routes such as that discovered for fungi that produce flavonoids (Zhang *et al.*, 2023). The synthesis of betalains from tyrosine branching of the shikimate pathway appears to be absent in *Serritaenia*, since the genes mapped to this pathway did not branch close to betalain-related genes of land plants (Supplementary Fig. S10). This is not surprising as betalains are only known from a single order of land plants (*Caryophyllales*) and some fungi (Stintzing and Schliemann, 2007; Babos *et al.*, 2011; Timoneda *et al.*, 2019).

However, *Serritaenia* expressed a number of genes that may encode enzymes that in land plants function in the core phenylpropanoid pathway and lignin biosynthesis. In agreement with previous studies (de Vries *et al*., 2021; Rieseberg *et al.*, 2022; Dadras *et al.*, 2023b), these land plant-based pathways are only fragmentarily recovered in streptophyte green algae, and some important enzymes such as phenylalanine ammonia-lyase (PAL), cinnamate 4-hydroxylase (C4H), 4-coumarate 3-hydroxylase (C3H), and ferulate-5-hydroxylase (F5H) could not be detected in the *Serritaenia* transcriptome. The lack of these enzymes in zygnematophytes (e.g. *Penium* and *Zygnema*) is known, but it is uncertain whether these algae perform the metabolic steps in question with different enzymes or evolved alternative pathways for phenylpropanoid synthesis. Overall, there is compelling evidence that zygnematophytes should be able to produce such compounds, as indicated by reports of the occurrence of flavonoids and phenylpropanoids in a wide range of green algae including chlorophytes (Aigner *et al.*, 2013; Pichrtová *et al.*, 2013; Goiris *et al.*, 2014; Jiao *et al.*, 2020). Specifically, we detected genes annotated as caffeoylshikimate esterase-like (CSE), hydroxylcinnamoyl-CoA:shikimate hydroxycinnamoyl transferase (HCT), cinnamyl-alcohol dehydrogenase (CAD), cinnamoyl-CoA reductase (CCR), 4-coumarate-CoA ligase (4CL), catechol-*O*-methyltransferase (COMT), caffeoyl-CoA *O*-methyltransferase (CCoAOMT), and class III peroxidases, most of which show significant regulation in response to UVR (Fig. 5A). For some candidates (e.g. CSE and HCT), the true activity remains unknown, as in our gene phylogenies they occupy distant positions to characterized plant enzymes (Supplementary Figs S11, S12), or are in a clade that also contains land plant genes with different activities (CAD and CCR; Supplementary Fig. S13; see also (de Vries *et al*. (2021). Furthermore, some core monolignol biosynthetic enzymes have related homologs that are involved in the primary metabolism, which makes functional annotation in phylogenetically distant organisms difficult (Weng and Chapple, 2010). Yet, the up-regulated acetyltransferase (HCT-annotated) and methyltransferase (CCoAOMT-annotated; Supplementary Fig. S14) sequences might encode proteins that act on hydroxycinnamic acids and thus are part of the phenylpropanoid pathway. The 4CL homolog branches with credible reference genes of other streptophytes as well (Supplementary Fig. S15). The CCoAOMT homologs, in particular, are interesting candidates as they show similarities in functional residues for ligand binding with plant enzymes and originated at the base of the *Phragmoplastophyta* (including *Charophyceae*, *Coleochaetophyceae*, *Zygnematophyceae*, and embryophytes) (de Vries *et al*., 2021). We still require both further transcriptomic profiling of zygnematophyte representatives and experimental studies on such protein candidates to shed light on their functions in unicellular green algae. Certainly, we cannot exclude that the enzymes encoded by some of the weakly annotated metabolic genes of *Serritaenia* are involved in other, as yet unknown pathways, which are not present in higher plants and, hence, not represented in current databases. These major discrepancies on the level of specialized metabolism clearly illustrate the deep evolutionary split between land plants and their closest algal relatives.

### Oxidative enzymes in the extracellular space

In plants, the products of the lignin-related phenylpropanoid pathway—the lignin precursors (monolignols, monolignol-ferulate ester, and flavone tricin)—are synthesized in the cytoplasm and transported to the apoplast (Barros *et al.*, 2015). Several mechanisms of transport, namely passive diffusion, active transport via G-family ATP-binding cassette (ABC) transporters, and secretion via vesicle–membrane fusion (especially for glucosylated monolignols), have been debated, but the relative contribution of these routes in the secretion of phenylpropanoids is still poorly understood (Barros *et al.*, 2015; Perkins *et al.*, 2019; Xin and Herburger, 2021). The ABCG transporters form a large, gene-rich family and transport various substrates, especially hydrophobic organic compounds (e.g. cutin monomers, lipids, wax components, and fatty acids), with varying specificity (Gräfe and Schmitt, 2021; Xin and Herburger, 2021). In streptophyte algae, such transporters are underexplored and uncharacterized, and their substrates might differ from those in land plants. Yet, these proteins may have important roles in the secretion of known and unknown algal specialized metabolites. We screened the transcriptome of *Serritaenia* for ABC transporters and found 28 genes that were up-regulated under the UVR treatment. Two candidates, both annotated as ABCG22, showed extreme up-regulation and expression (Fig. 5B; Supplementary Table S13). Interestingly, transporters of this family have been suspected to be involved in lignification in *A. thaliana*, as they were co-expressed with AtABCG29, which evidently transports monolignols (Alejandro *et al.*, 2012), and other lignification-associated factors (Takeuchi *et al.*, 2018a). In fact, several homologs of the ABCG transporters from *A. thaliana* have been associated with lignification and the transport of phenylpropanoids on the basis of expression patterns, for example ABCG30, ABCG33, ABCG34, and ABCG37 (Takeuchi *et al.*, 2018b). However, experimental evidence for most plant ABCG transporters is still lacking and the evolutionary significance of their diversity is unknown. As already proposed by plant biologists (Xin and Herburger, 2021), the study of algal ABC transporters might be an informative, complementing approach. The two ABCG22 homologs found to be strongly expressed during UVR-induced pigment production in *Serritaenia* might be interesting candidates.

The final part of the lignin-related phenylpropanoid pathway in plants is the oxidative polymerization of lignin precursors in the apoplast. This reaction is performed by extracellular enzymes such as heme-containing peroxidases of class III (Marjamaa *et al.*, 2009; Fagerstedt *et al.*, 2010). These enzymes are secreted into the extracellular space and catalyze the reduction of H_2_O_2_ by transferring electrons from various donor molecules, such as phenolic compounds, lignin precursors, auxin, or secondary metabolites, and can also function as generators of ROS (Weng and Chapple, 2010; Shigeto and Tsutsumi, 2016). The KEGG annotations of the *Serritaenia* transcriptome revealed the presence of a class III peroxidase (Fig. 5A), which turned out to be the gene with the highest up-regulation (log2FC=10.7) in the transcriptome. The hypothetical protein of 340 amino acids contains a signal peptide (likelihood 0.99) and is predicted to be localized in the extracellular space (probability 0.8) according to SignalP 6.0 (Teufel *et al.*, 2022) and DeepLoc 2.0 (Thumuluri *et al.*, 2022), respectively. Blast annotations with the RedOxiBase dataset (Savelli *et al.*, 2019) confirmed its affinity for plant class III peroxidases; the three closest hits from *A. thaliana* were AtPrx30, AtPrx53, and AtPrx54. Despite the relatively low sequence identity with plant homologs (<50%), the *Serritaenia* peroxidase contains residues predicted to bind heme, calcium ions, and the substrate ferulic acid (Fig. 5C). We also performed an *in silico* structure prediction with I-TASSER (Yang and Zhang, 2015), which confirmed the heme- and calcium-binding sites (C-scores 0.77 and 0.03, respectively). The most similar hits from the RCSB protein data bank were a highly glycosylated peroxidase from the royal palm tree *Roystonea regia* [RPTP (3HDL); TM score 0.865] and peroxidase A2 from *A. thaliana* [AtPrx53 (1PA2); TM score 0.862]. The RPTP is an extracellular enzyme with superior stability (Zamorano *et al.*, 2008), which showed high activity on ferulic acid, a central phenolic compound in the phenylpropanoid pathway (Sakharov *et al.*, 2001, 2002). The peroxidase A2 (AtPrx53) from *A. thaliana* was suggested to have a role in lignification, as this protein was highly expressed in lignifying cells and tissues, and the substrate-binding site was predicted to bind and oxidize lignin precursors, especially *p*-coumaroyl and coniferyl alcohols (Ostergaard *et al.*, 2000). However, class III peroxidases are involved in various biological processes and have a broad substrate spectrum. Hence, it is not possible to assign specific functions on the basis of annotations or sequence homology.

Class III peroxidases have already been detected in streptophyte green algae (Buschmann and Holzinger, 2020; Mbadinga Mbadinga *et al.*, 2020), but the algal homologs are still vastly underexplored and uncharacterized. We collected peroxidase sequences from 23 streptophyte algae (including 15 zygnematophytes) and performed phylogenetic inferences to assess the diversity of these proteins and to understand the evolution of the peroxidases of *Serritaenia* (Fig. 5D). Even though the deeper branches are not well resolved due to limited phylogenetic signal, we observed a number of algal peroxidase clades with pronounced diversification of these proteins in several taxa, especially in the genera *Chaetosphaeridium* and *Coleochaete* (Supplementary Fig. S16). The facts that (i) the algal clades are nested within the embryophyte peroxidases and (ii) the peroxidases of a single algal species occur at different positions in the tree, suggest that a certain degree of diversification happened well before the evolution of land plants. The class III peroxidases of the zygnematophyte order *Serritaeniales* form a single well-supported clade, reflecting the phylogeny of the organisms (Hess *et al.*, 2022). We added information of three other *Serritaenia* strains (two sequenced in this study), which represent the phylogenetic diversity of the genus. The peroxidases of the *Serritaenia* strains were closely related and stem from a single ancestor. According to our phylogeny, there were up to four potential gene duplication events, some of which may have occurred before the diversification of the *Serritaenia* species. However, only one of the four homologs (StesPrx01) found in *S. testaceovaginata* displayed massive up-regulation and pronounced expression upon UVR exposure, indicating that the class III peroxidases differ in function and/or biological relevance (Fig. 5D). This is the first report of UVR-related up-regulation of a secreted class III peroxidase in a streptophyte alga, similar to the reaction of vascular plants such as *Nicotiana tabacum* (Rácz *et al.*, 2018), *Helianthus annuus* (Yannarelli *et al.*, 2006), and *A. thaliana* (Rao *et al.*, 1996). This finding, along with some highly up-regulated multicopper oxidase domain-containing proteins (Supplementary Table S14), points to increased oxidative reactions in the cell wall and/or mucilage of *Serritaenia* when exposed to UVR. In land plants, such enzymes perform various important roles, including ROS scavenging, signaling, and the polymerization of extracellular phenolics (McCaig *et al.*, 2005; Shigeto and Tsutsumi, 2016). Just recently, the multicopper oxidase-like enzymes of the SKS family have been shown to be required for coumaroylation of sporopollenin in pollen (Xu *et al.*, 2023). While phenolic polymers, referred to as ‘lignin-like substances’, have been detected in other streptophyte green algae (*Coleochaete* and *Nitella*; Delwiche *et al.*, 1989; Ligrone *et al.*, 2008), there is currently no evidence for them in the zygnematophytes. Given the pronounced regulation of phenylpropanoid-related enzymes, ABCG transporters, and oxidative enzymes predicted in the extracellular environment, it might well be that polymeric phenylpropanoids enriched in the algal mucilage fulfill the remarkable sunscreen function in *Serritaenia*. The connection between extracellular (cuticular) phenolics and the ‘pre-lignin’ pathway was already established for bryophytes (Renault *et al.*, 2017). With this study, we provide expression data and sequence information of UVR-responsive candidate genes, that will enable us to experimentally test the role of such a ‘pre-lignin’ pathway and its products in the closest algal relatives of land plants.

### Conclusion

With comparative transcriptomics, this study sheds some light on the cellular changes of a non-model zygnematophyte with a unique sunscreen mechanism. Overall, the data suggest that fundamental processes such as photosynthesis and light/UVR perception are relatively conserved and react similarly to what is known from land plants. However, the plant-based specialized metabolism was only fragmentarily recovered, which reflects the large evolutionary split between plants and zygnematophytes, and points to a major lack of knowledge concerning algal metabolic processes. Two important specialized metabolite pathways (flavonoid biosynthesis, including flavones and flavonols, or anthocyanins), which in plants have significant roles in UVR protection, do not appear to play a role in *Serritaenia*’s reaction to UV-B. Instead, we discovered marked regulation of enzymes mapped on the shikimate and phenylpropanoid pathway, potential cross-membrane transporters of phenolics, and oxidative enzymes targeted to the extracellular space. Plant homologs of the latter are known to act on extracellular phenolics to form polymeric lignin in the apoplast, which is mainly associated with the mechanical properties of plant tissues. However, given the substrate promiscuity of class III peroxidases and the extent of uncharacterized homologs, these enzymes might produce many more extracellular compounds of varying function in plants and algae. The extracellular pigment of *Serritaenia* is surprisingly resistant to solvents and hydrolysis, and, despite its different function, might share a common origin with plant lignins.

## Supplementary data

The following supplementary data are available at *JXB* online.

Fig. S1. RNA samples used for sequencing.

Fig. S2. UpSet plot of knumber annotations.

Fig. S3. KEGG pathway nucleotide excision repair.

Fig. S4. KEGG pathway base excision repair.

Fig. S5. KEGG pathway mismatch repair.

Fig. S6. GO terms enriched in down-regulated genes.

Fig. S7. Phylogenetic tree of CHS.

Fig. S8. Phylogenetic tree of CHALCONE ISOMERASE (CHI) and CHI-Like (CHIL).

Fig. S9. Phylogenetic tree of UGT.

Fig. S10. Phylogenetic tree of ligB genes.

Fig. S11. Phylogenetic tree of caffeoylshikimate esterases.

Fig. S12. Phylogenetic tree of BAHD acyltransferases.

Fig. S13. Phylogenetic tree of NAD(P)H-dependent reductases (CAD and CCR-like).

Fig. S14. Phylogenetic tree of *O*-methyltransferases.

Fig. S15. Phylogenetic tree of 4-coumaroyl CoA:ligase (4CL).

Fig. S16. Phylogenetic tree of class III peroxidases (PRXIII) from streptophyte representatives (algae, bryophytes, ferns, and flowering plants).

Table S1. Recipe of algal culture medium KW.

Table S2. Streptophyte green algal transcriptomes and genomes screened for class III peroxidases.

Table S3. Enriched gene ontology term ‘response to UV’ (GO:0009411) of up-regulated genes.

Table S4. Heat shock proteins.

Table S5. BlastP result of selected jasmonate pathway-related proteins.

Table S6. Potential homologs of jasmonate pathway-related proteins in *S. testaceovaginata*.

Table S7. Photosynthesis-related proteins.

Table S8. Anti-ROS factors.

Table S9. Photoreceptors and associated proteins.

Table S10. BlastP result of scytonemin biosynthesis-related proteins.

Table S11. BlastP result of mycosporine-like amino acids biosynthesis-related proteins.

Table S12. Shikimate pathway and specialized metabolism.

Table S13. ABC transporters.

Table S14. Top 50 up-regulated genes.

Dataset S1. Trimmed alignment of class III peroxidases.

erae131_suppl_Supplementary_Figures_S1-S16_Tables_S1-S2

erae131_suppl_Supplementary_Tables_S3-S14

erae131_suppl_Supplementary_Dataset_S1

## Data Availability

The data that support the findings of this study are openly available. The RNA-seq reads can be found on ArrayExpress at https://www.ebi.ac.uk/biostudies/arrayexpress, accession E-MTAB-13832. The transcriptome assemblies can be found on ENA (European Nucleotide Archive) at https://www.ebi.ac.uk/ena/browser/home, accession PRJEB72628. Gene expression data and functional annotations of transcripts are publicly available in Zenodo at https://doi.org/10.5281/zenodo.10680943.
