# Supplementary material for: Comparative transcriptomics elucidates the cellular responses of an aeroterrestrial zygnematophyte to UV radiation
Source: J Exp Bot. 2024 Mar 23;75(11):3624–42. doi: 10.1093/jxb/erae131 (PMC11156808; doi:10.1093/jxb/erae131)
Supplement: erae131_suppl_Supplementary_Figures_S1-S16_Tables_S1-S2 [file erae131_suppl_supplementary_figures_s1-s16_tables_s1-s2.pdf]

Supplementary Data

Article title: Comparative transcriptomics illuminates the cellular responses of an aeroterrestrial zygnematophyte to UV radiation

Authors: Anna Busch, Jennifer V. Gerbracht, Kevin Davies, Ute Hoecker, Sebastian Hess

Fig. S1 RNA samples used for sequencing. RNA denaturing gel of isolated RNA from *Serritaenia testaceovaginata* under condition 1 (NK) and condition 2 (UV) in triplicates.

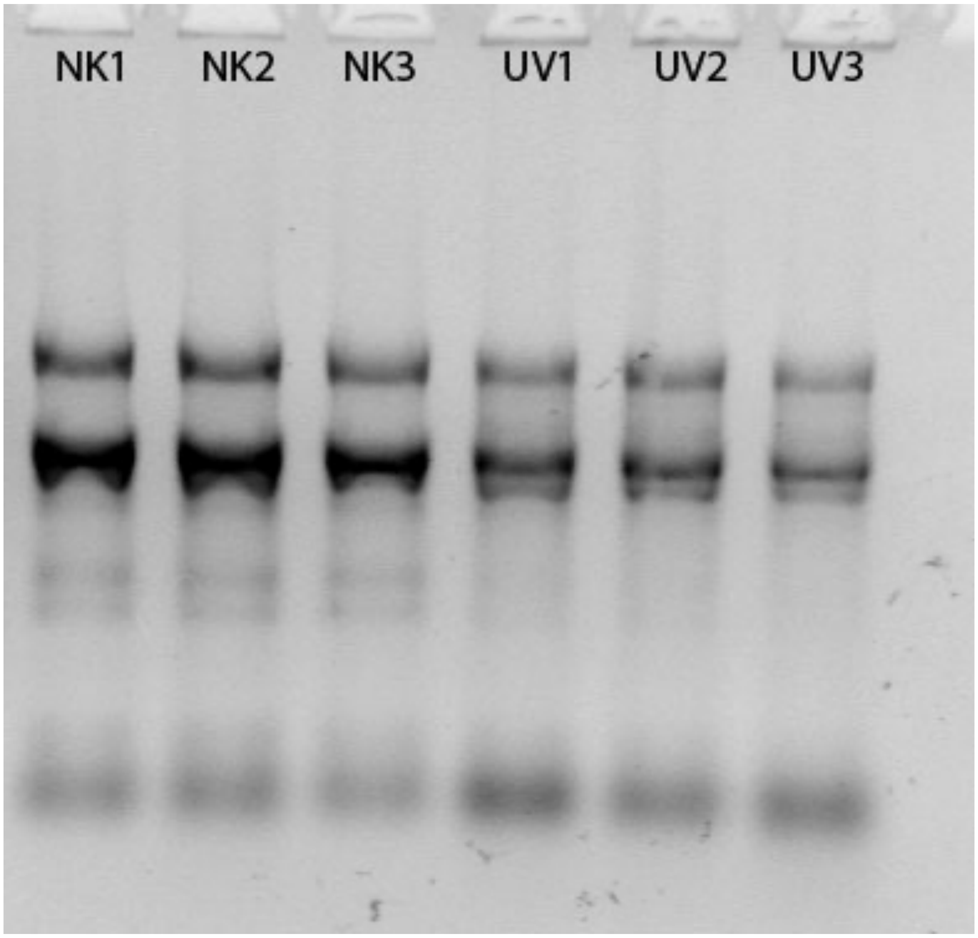

Fig. S2 UpSet plot of knumber annotations. UpSet plot showing the number of ORFs (intersection size) annotated by the indicated annotation tools and databases and their combinations.

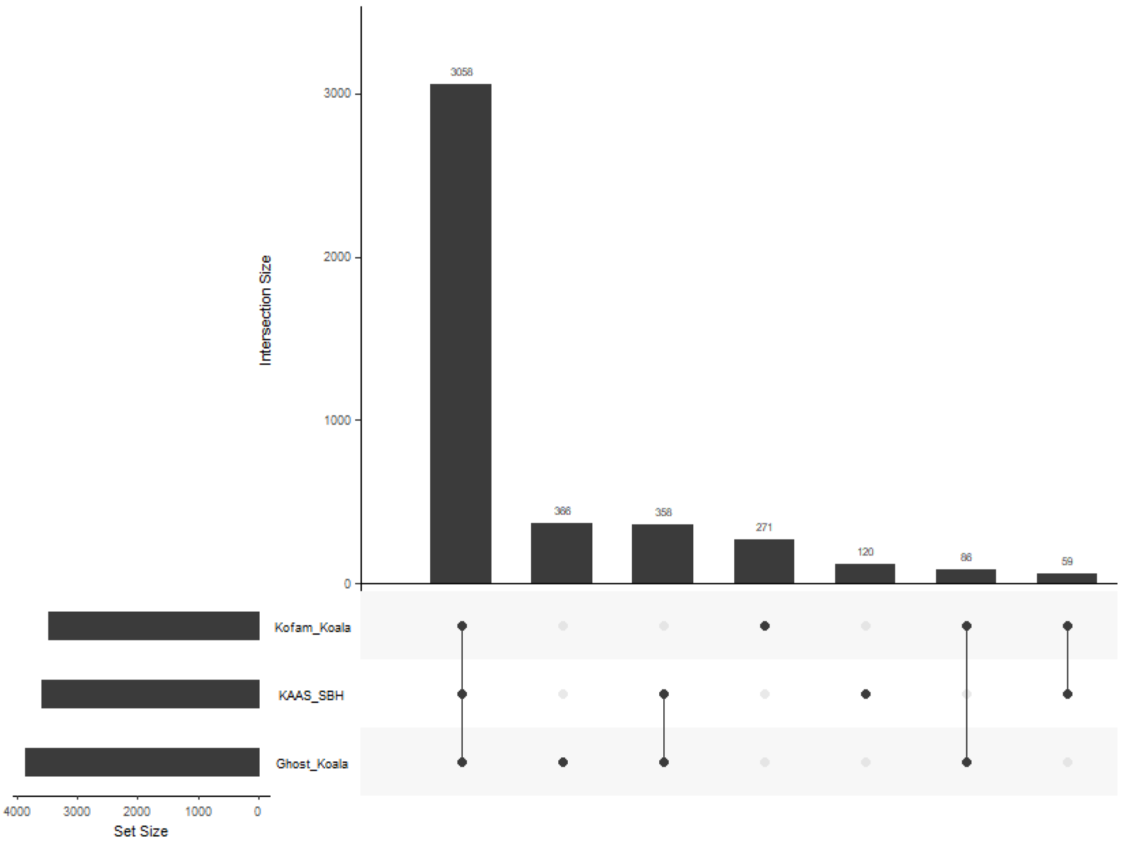

Fig. S3 KEGG pathway nucleotide excision repair. Presence and regulation in *S. testaceovaginata* are indicated by the color of the boxes.

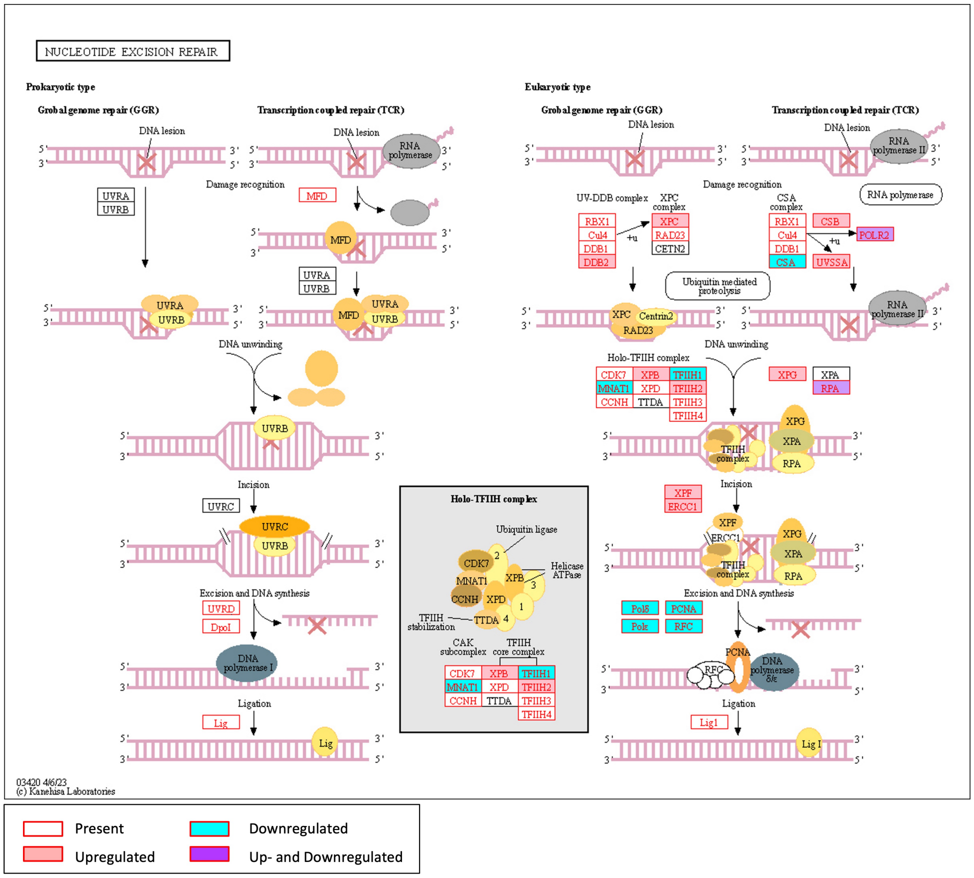

Fig. S4 KEGG pathway base excision repair. Presence and regulation in *S. testaceovaginata* are indicated by the color of the boxes.

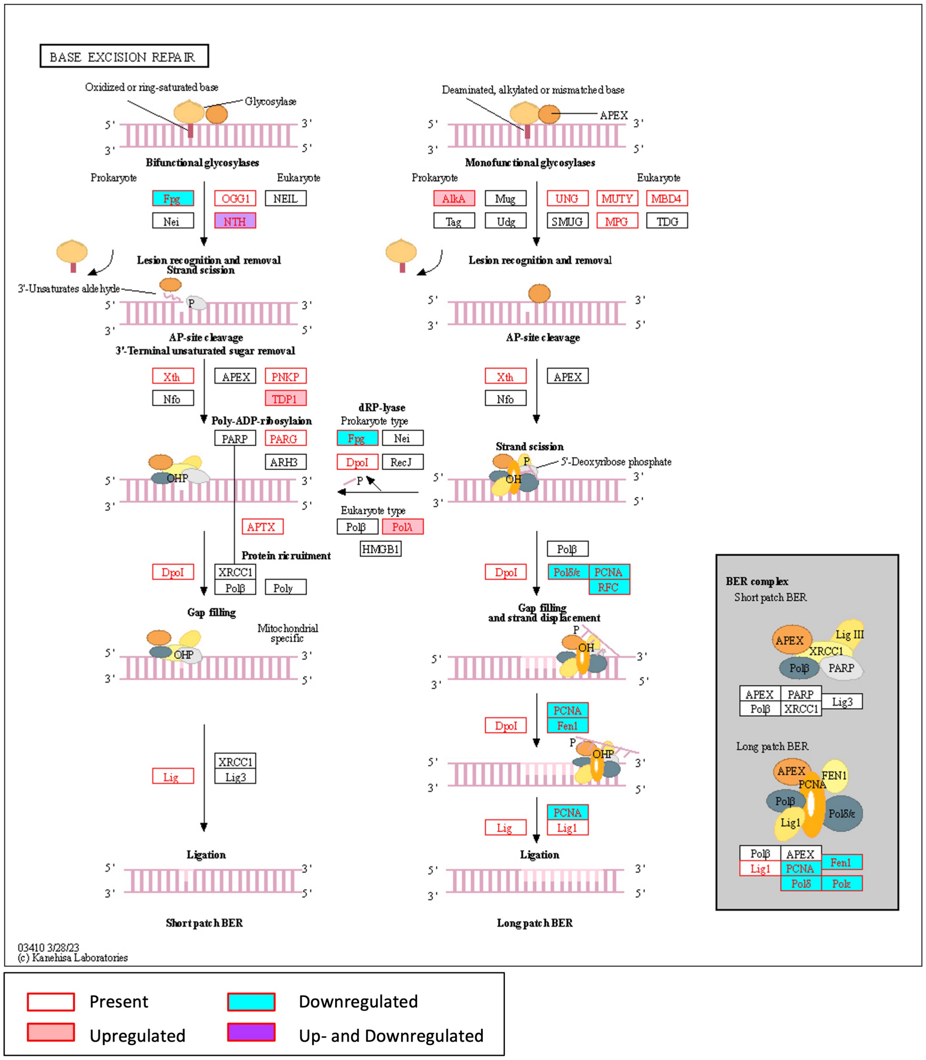

Fig. S5 KEGG pathway mismatch repair. Presence and regulation in *S. testaceovaginata* are indicated by the color of the boxes.

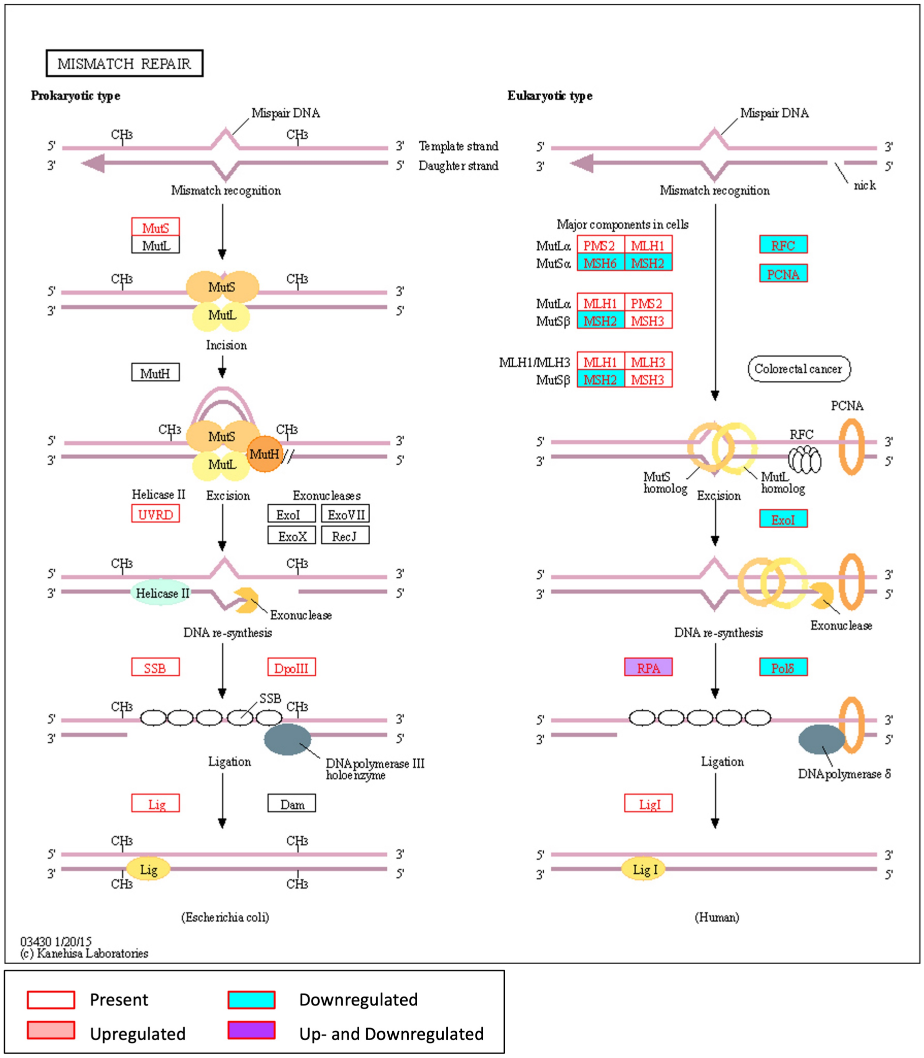

Fig. S6 GO terms enriched in downregulated genes.

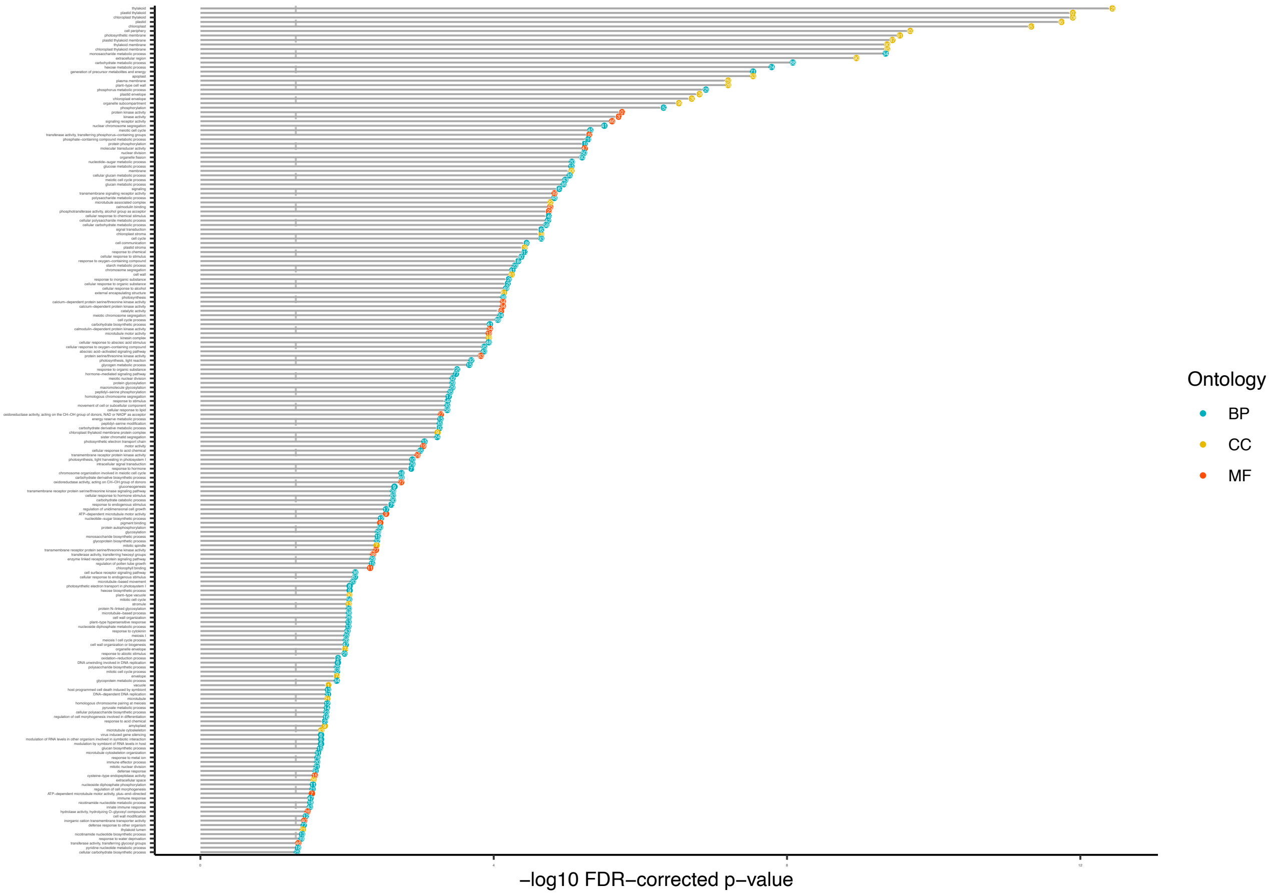

Fig. S7 Phylogenetic tree of CHS.

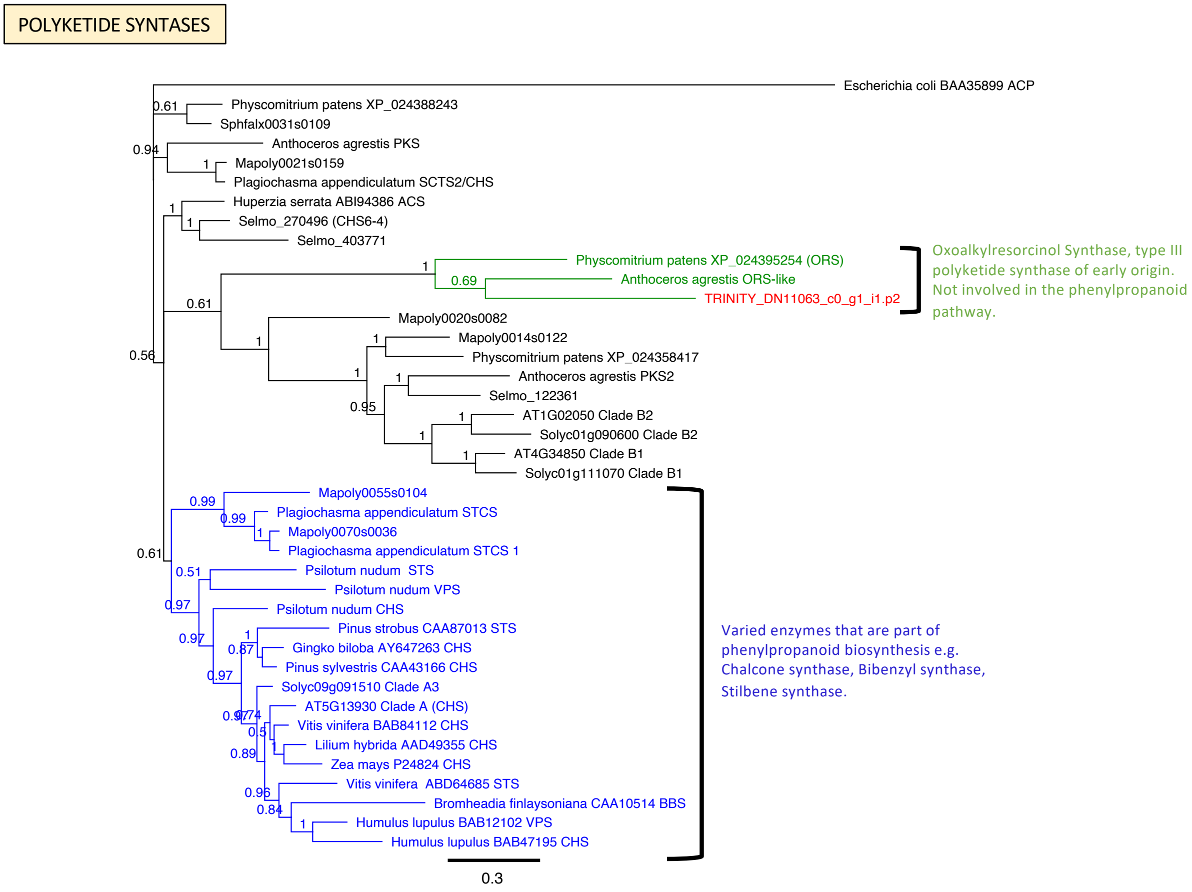

Fig. S8 Phylogenetic tree of CHALCONE ISOMERASE (CHI) and CHI-Like (CHIL).

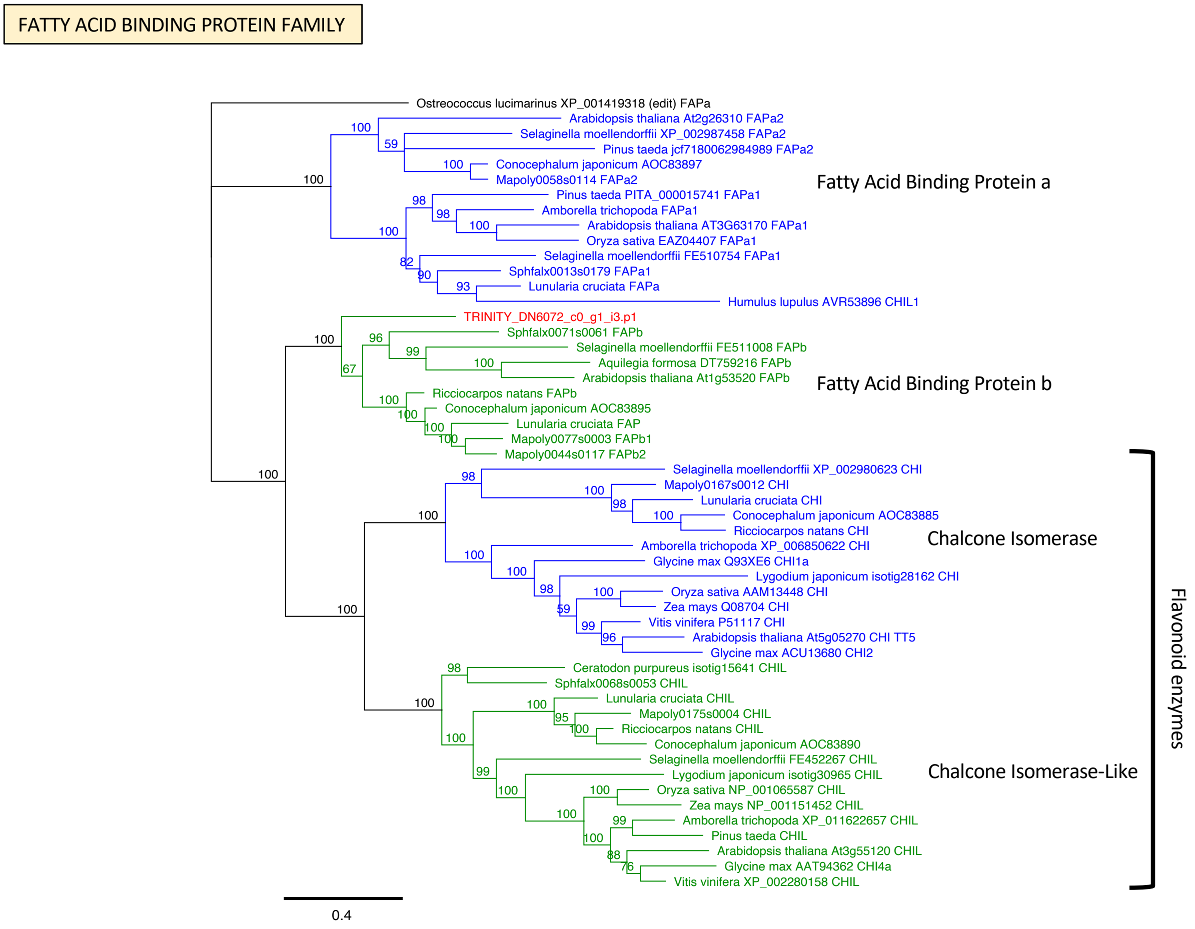

Fig. S9 Phylogenetic tree of UGT.

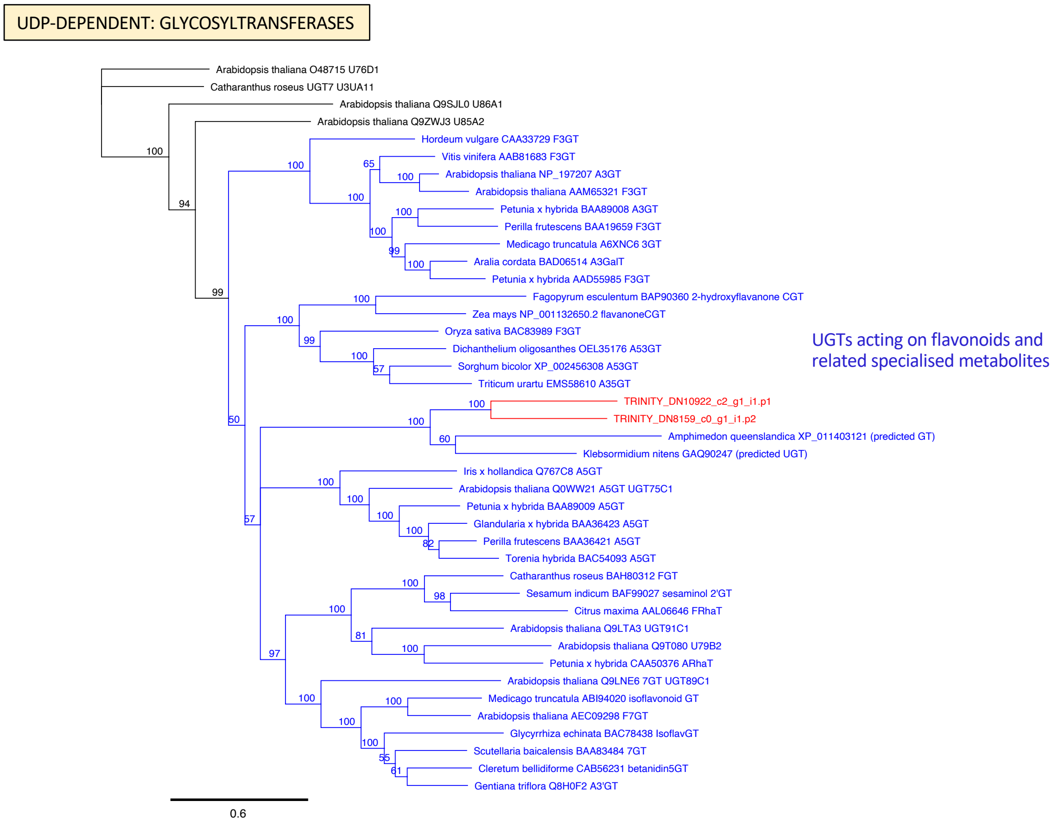

Fig. S10 Phylogenetic tree of ligB genes.

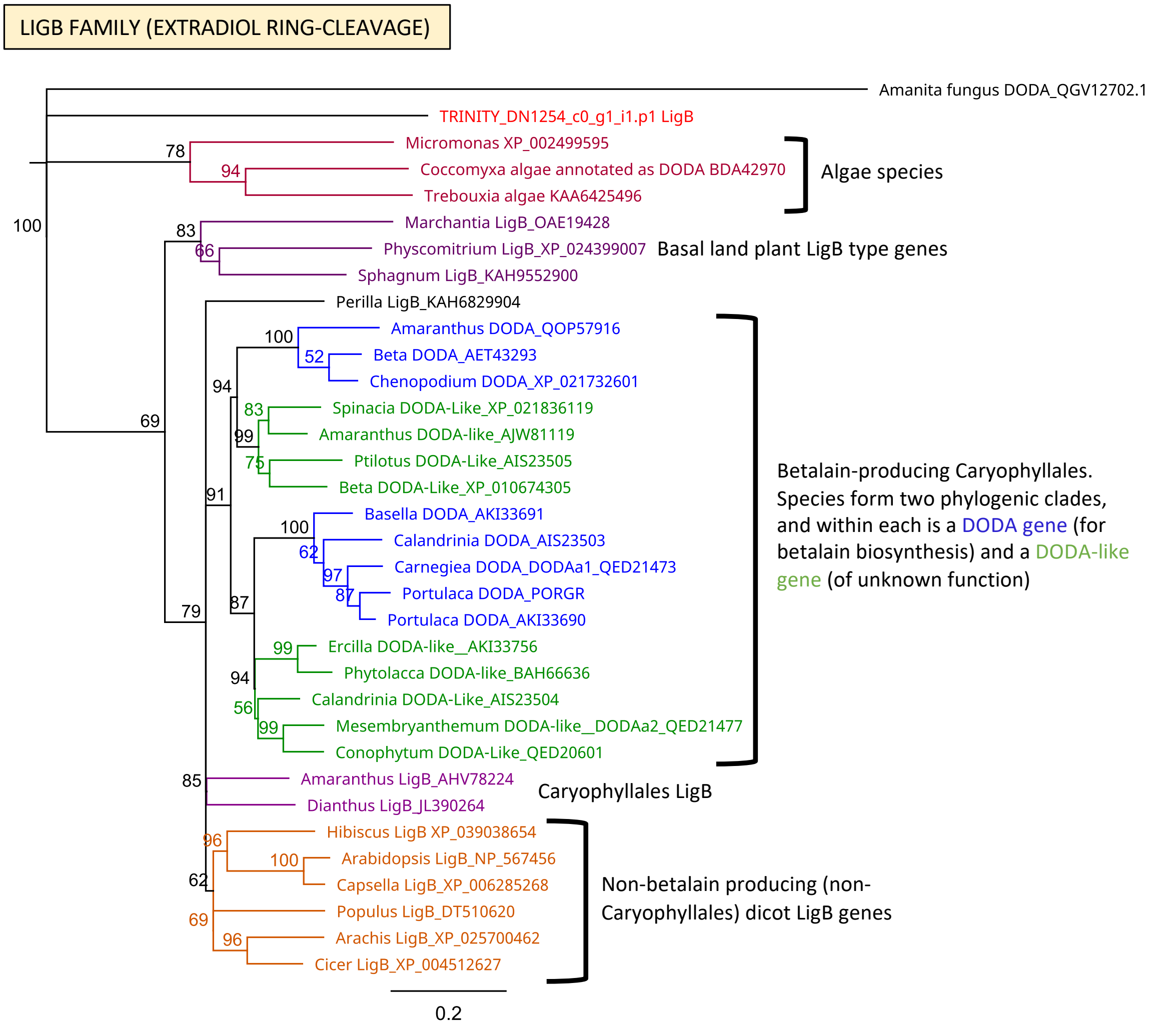

Fig. S11 Phylogenetic tree of caffeoylshikimate esterases.

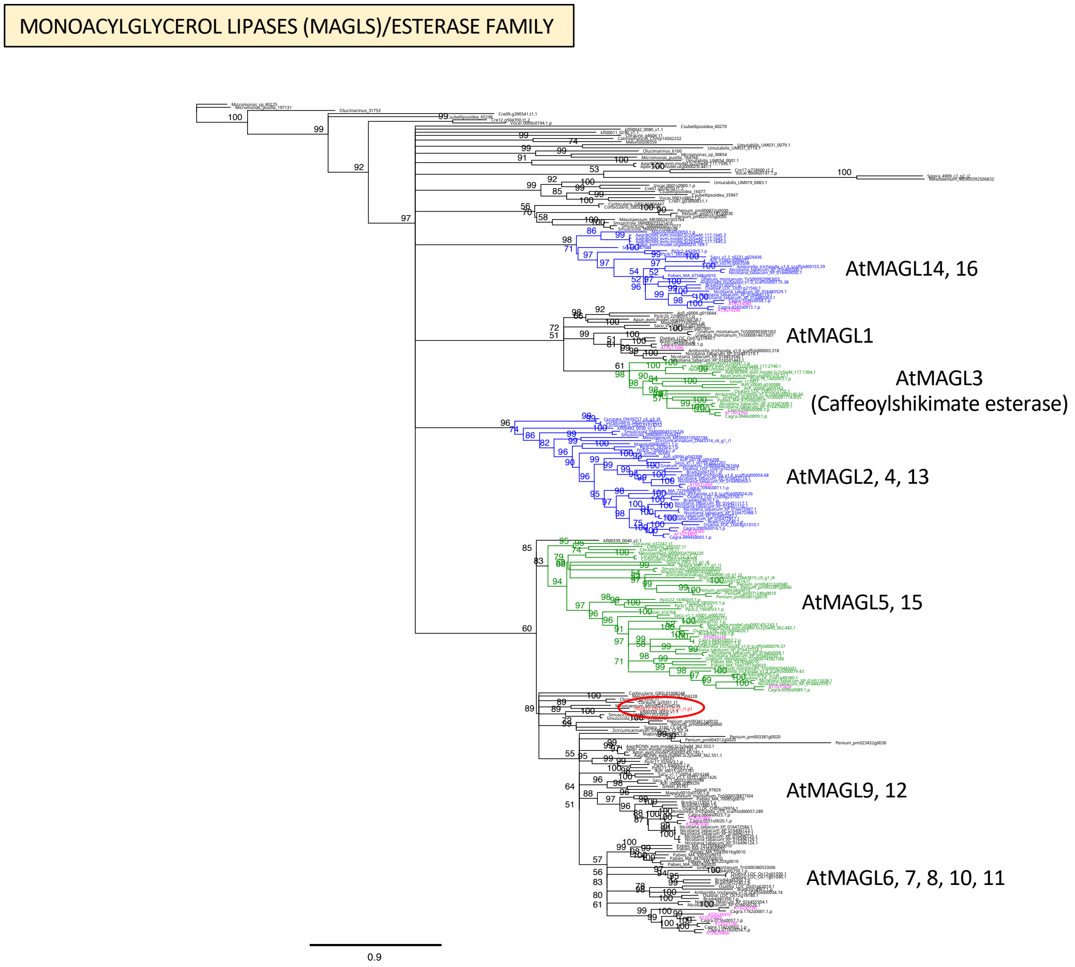

Fig. S12 Phylogenetic tree of BAHD acyltransferases.

HCT BADH Family

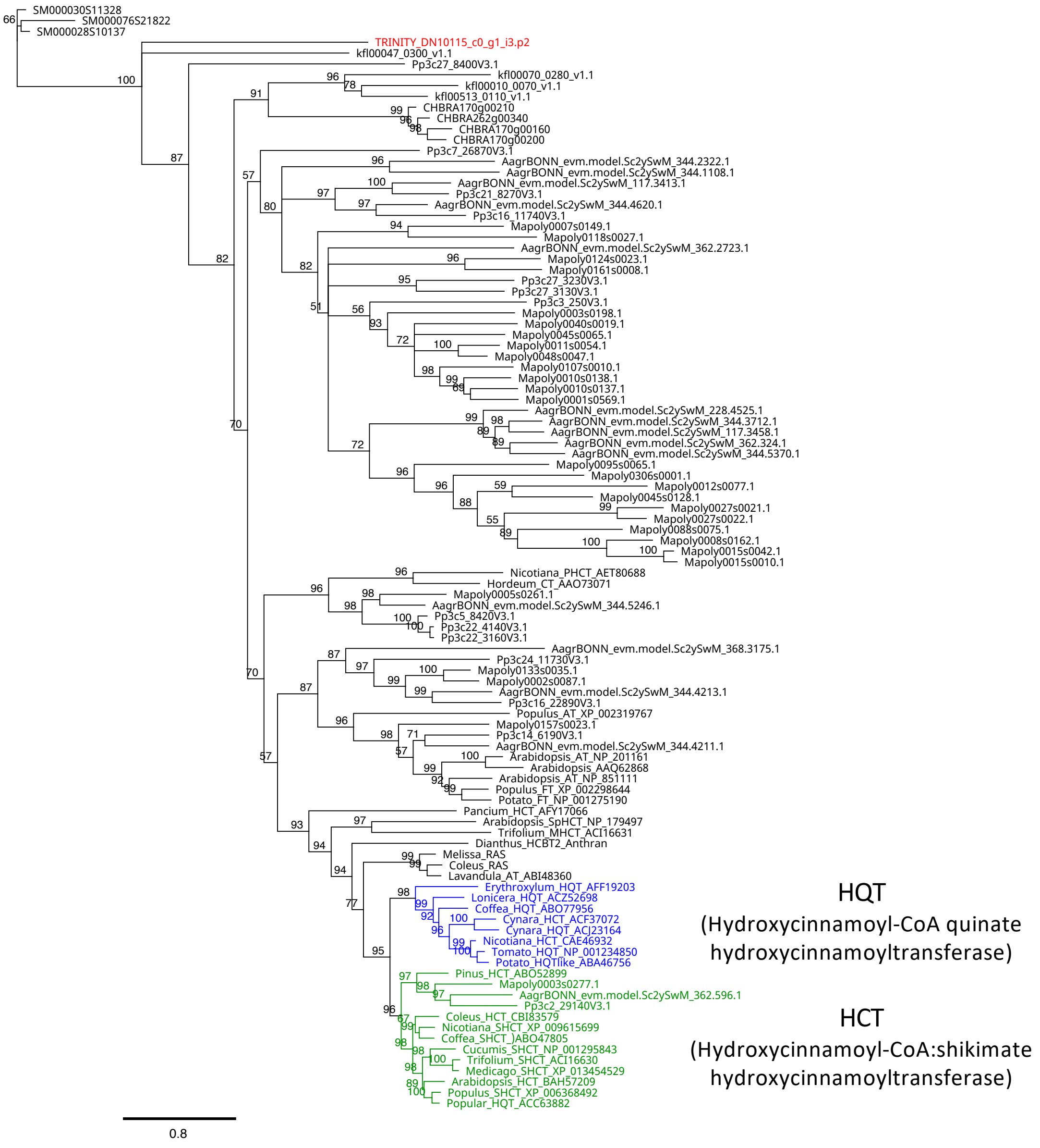

Fig. S13 Phylogenetic tree of NAD(P)H-dependent reductases (CAD and CCR-like).

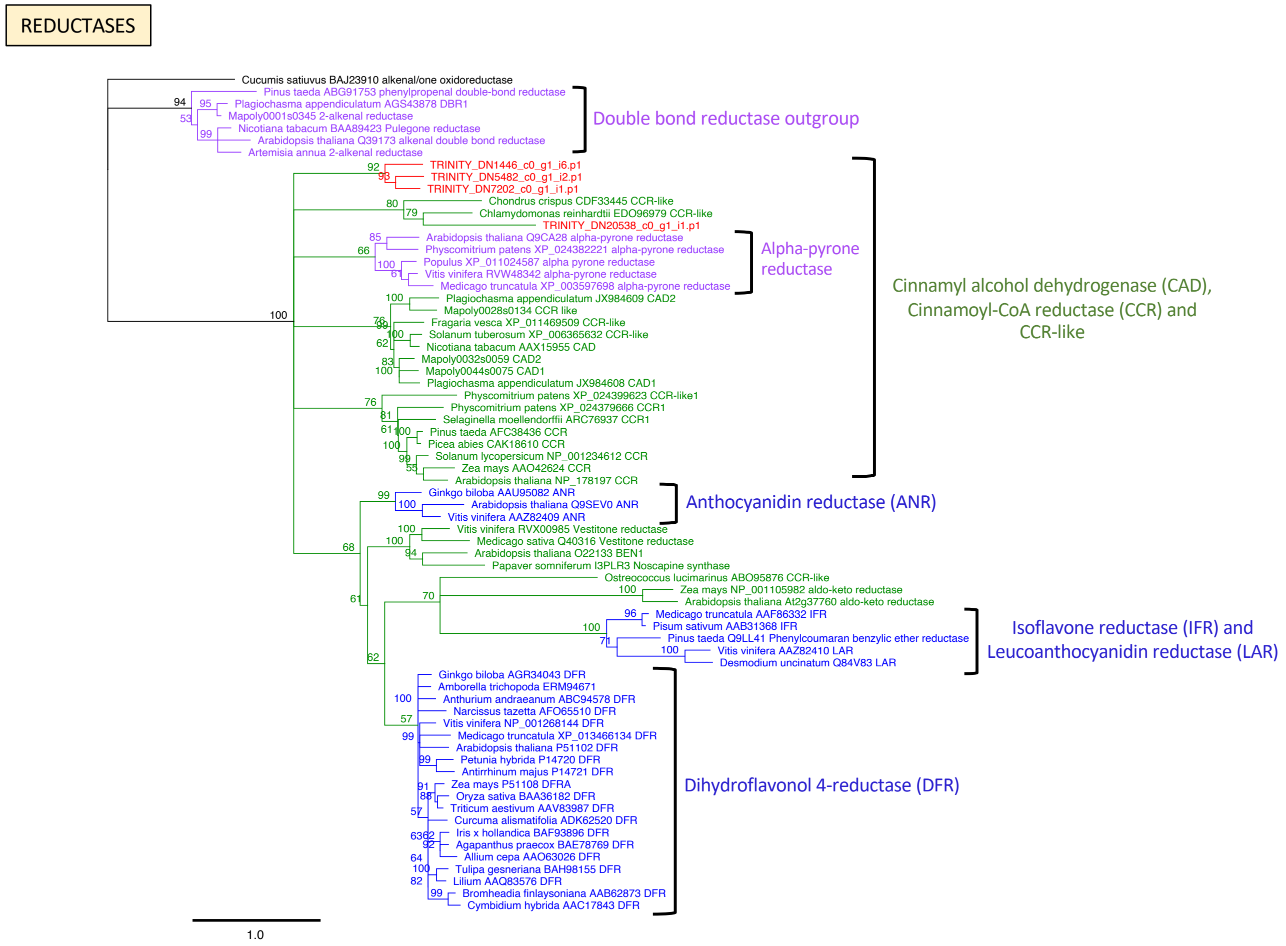

Fig. S14 Phylogenetic tree of *O*-methyltransferases.

O-METHYLTRANSFERASES

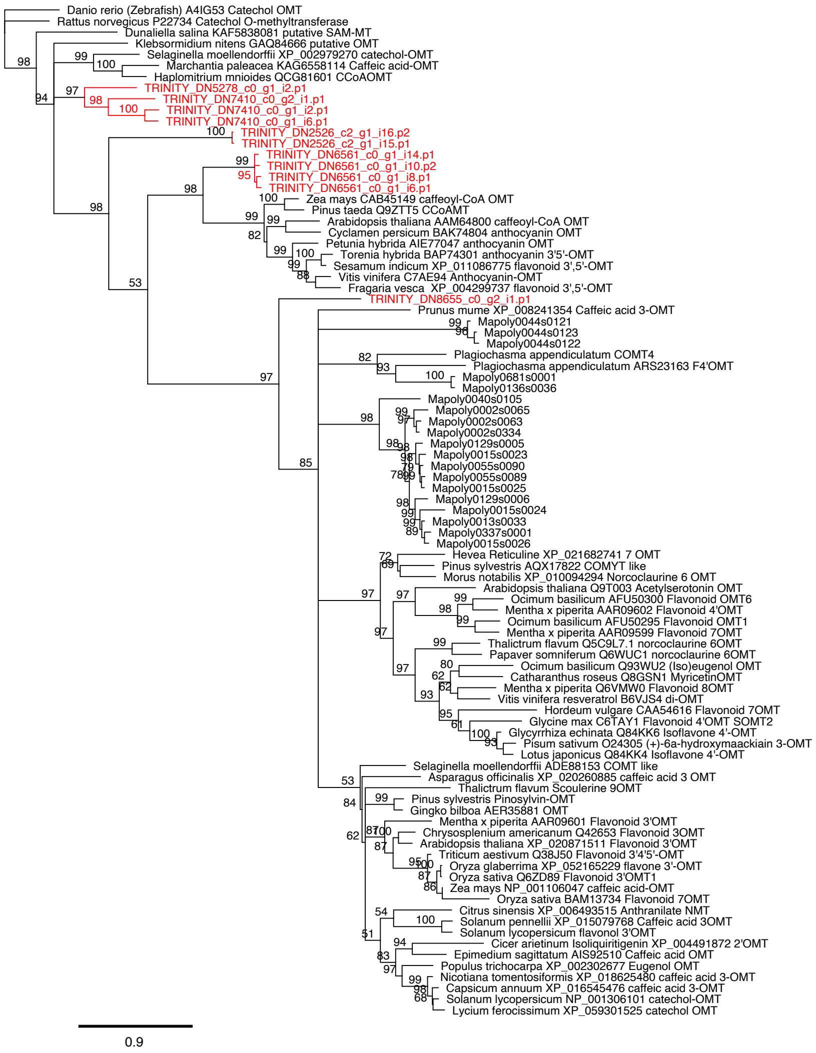

Fig. S15 Phylogenetic tree of 4-Coumaroyl CoA: ligase (4CL).

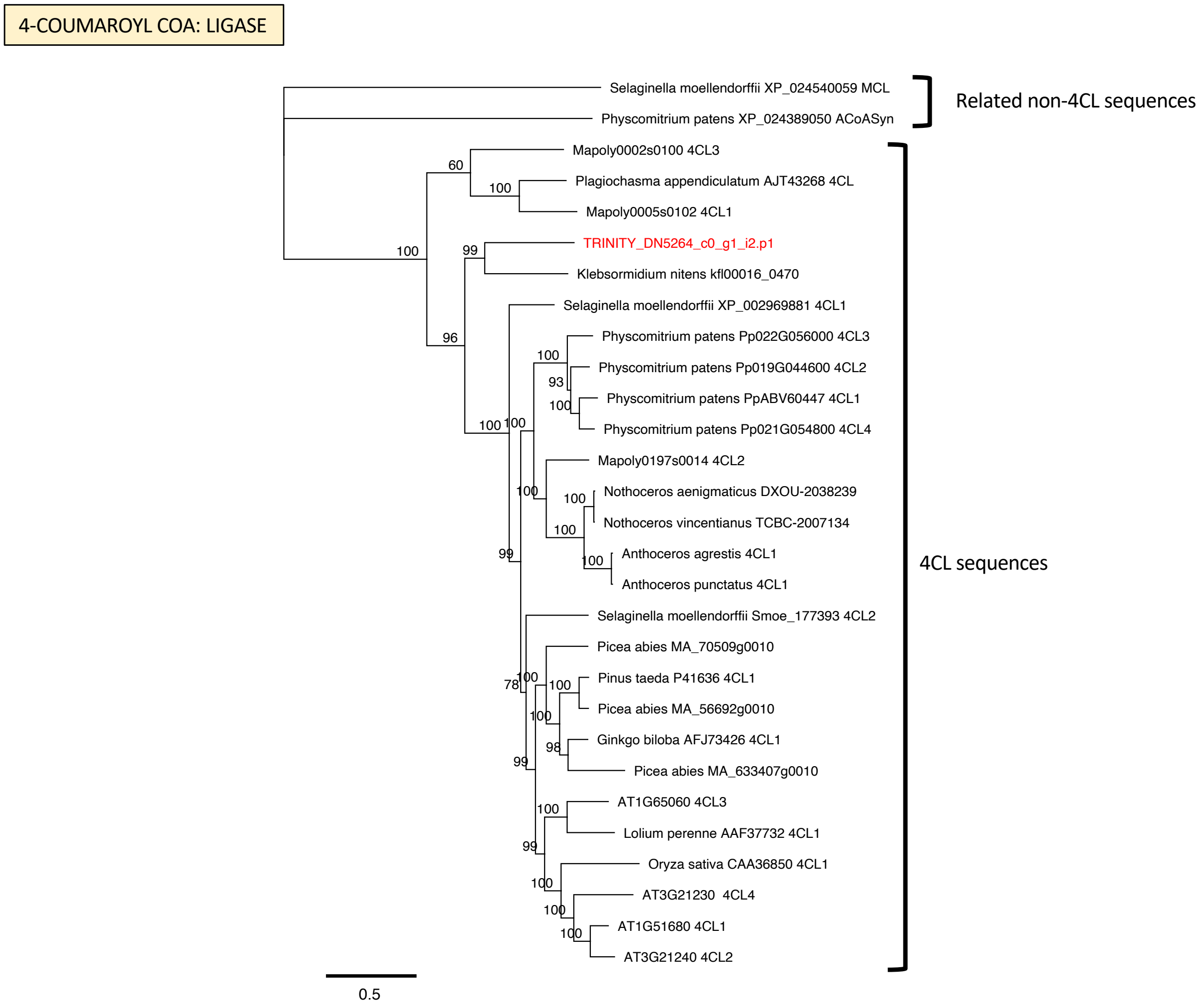

Fig. S16 Phylogenetic tree of class III peroxidases (PRXIII) from streptophyte representatives (algae, bryophytes, ferns and flowering plants). Sequences of streptophyte algae are highlighted in red and ultrafast bootstrap values are shown at the branches. Scale bar = number of expected substitutions per site. * annotation stems from RedOxiBase (samples were wrongly annotated in the 1kp dataset and might originate from *Zygnema* sp.)

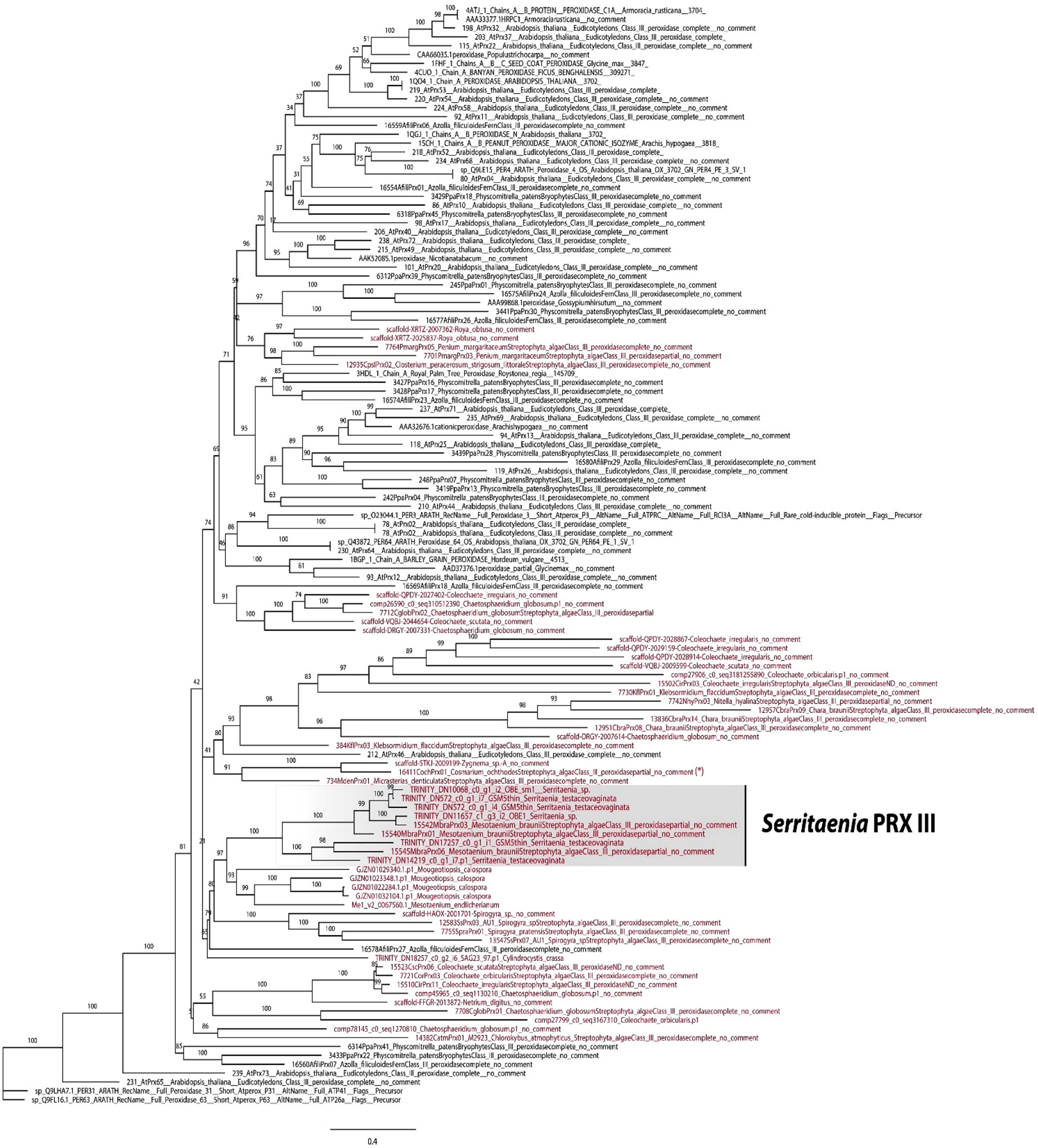

Table S1 Recipe of algal culture medium KW. One milliliter of each stock solution is added to one liter of demineralized water. The pH should be around 6.

Components	Stock solution
HEPES	238.1 g/l dH_2O
KNO_3	100 g/ l dH_2O
$MgSO_4$ x 7 H_2O	20 g/l dH_2O
NaH_2PO_4 x H_2O	0.69 g/50 ml
Na_2HPO_4 x 2 H_2O	0.89 g /50 ml
$CaCl_2$ x 2 H_2O	14.7 g/l dH_2O
P-II Metals stock solution<br>EDTA (Titriplex III)<br>H_3BO_3<br>$MnCl_2$ x 4 H_2O<br>$ZnSO_4$ x 7 H_2O<br>$CoCl_2$ x 6 H_2O	 3.00 g/l dH2O<br>1.14 g/l dH2O<br>144.00 mg/l dH2O<br>21.00 mg/l dH2O<br>4.00 mg/l dH2O
Fe-EDTA stock solution<br>EDTA (Titriplex II)<br>$FeSO_4$ x 7 H_2O<br>1N KOH	 5.22 g / l dH2O<br>4.98 g / l dH2O<br>54.00 ml / l dH2O

Table S2 Streptophyte green algal transcriptomes and genomes screened for class III peroxidases.

Strain	Data type	Source of data	Data availability
Penium margaritaceum AEKF	Transcriptome	1kp project	https://www.onekp.com/public_data.html
Chaetosphaeridium globosum DRGY	Transcriptome	1kp project	https://www.onekp.com/public_data.html
Netrium digitus FFGR	Transcriptome	1kp project	https://www.onekp.com/public_data.html
Klebsormidium subtile FQLP	Transcriptome	1kp project	https://www.onekp.com/public_data.html
Spirogyra sp. HAOX	Transcriptome	1kp project	https://www.onekp.com/public_data.html
Cylindrocystis cushleckae JOJQ	Transcriptome	1kp project	https://www.onekp.com/public_data.html
Spirotaenia minuta NNHQ	Transcriptome	1kp project	https://www.onekp.com/public_data.html
Coleochaete irregularis QPDY	Transcriptome	1kp project	https://www.onekp.com/public_data.html
Cosmarium ochthodes STKJ	Transcriptome	1kp project	https://www.onekp.com/public_data.html
Coleochaete scutata VQBJ	Transcriptome	1kp project	https://www.onekp.com/public_data.html
Mesotaenium endlicherianum WDCW	Transcriptome	1kp project	https://www.onekp.com/public_data.html
Roya obtusa XRTZ	Transcriptome	1kp project	https://www.onekp.com/public_data.html
Cylindrocystis brebissonii YOXI	Transcriptome	1kp project	https://www.onekp.com/public_data.html
Mougeotia sp. ZRMT	Transcriptome	1kp project	https://www.onekp.com/public_data.html
Mesotaenium endlicherianum	Genome	Cheng et al., 2019	https://figshare.com/articles/dataset/Genomes_of_subaerial_Zygnematophyceae_provide_insights_into_land_plant_evolution/9911876/1
Spirogloea muscicola	Genome	Cheng et al., 2019	https://figshare.com/articles/dataset/Genomes_of_subaerial_Zygnematophyceae_provide_insights_into_land_plant_evolution/9911876/1
Chaetosphaeridium globosum SAG26.98	Transcriptome	Cooper and Delwiche, 2016	https://figshare.com/articles/dataset/Green_algal_transcriptomes_for_phylogenetics_and_comparative_genomics/1604778
Coleochaete orbicularis	Transcriptome	Cooper and Delwiche, 2016	https://figshare.com/articles/dataset/Green_algal_transcriptomes_for_phylogenetics_and_comparative_genomics/1604778
Klebsormidium flaccidum UTEX 321	Transcriptome	Cooper and Delwiche, 2016	https://figshare.com/articles/dataset/Green_algal_transcriptomes_for_phylogenetics_and_comparative_genomics/1604778
Mougeotia scalaris SAG164.80	Transcriptome	Cooper and Delwiche, 2016	https://figshare.com/articles/dataset/Green_algal_transcriptomes_for_phylogenetics_and_comparative_genomics/1604778
Nitella mirabilis transcriptomes of lower and upper tissues	Transcriptome	Cooper and Delwiche, 2016	https://figshare.com/articles/dataset/Green_algal_transcriptomes_for_phylogenetics_and_comparative_genomics/1604778

Penium margaritaceum SAG22.82	Transcriptome	Cooper and Delwiche, 2016	https://figshare.com/articles/dataset/Green_algal_transcriptomes_for_phylogenetics_and_comparative_genomics/1604778
Spirogyra pratensis UTEX 921	Transcriptome	Cooper and Delwiche, 2016	https://figshare.com/articles/dataset/Green_algal_transcriptomes_for_phylogenetics_and_comparative_genomics/1604778
Spirogyra sp. Transcriptome Au1	Transcriptome	Cooper and Delwiche, 2016	https://figshare.com/articles/dataset/Green_algal_transcriptomes_for_phylogenetics_and_comparative_genomics/1604778
Serritaenia sp. OBE.sm1	Transcriptome	This study	https://www.ebi.ac.uk/ena/browser/view/PRJEB72628
Serritaenia sp. OBE.1	Transcriptome	This study	https://www.ebi.ac.uk/ena/browser/view/PRJEB72628
Cylindrocystis crassa SAG23.97	Transcriptome	This study	https://www.ebi.ac.uk/ena/browser/view/PRJEB72628
Mesotaenium endlicherianum SAG12.97	Transcriptome	Dadras et al., 2022	https://mesotaenium.uni-goettingen.de/download.html
Mougeotiopsis calospora MZCH580	Transcriptome	Hess et al., 2022	https://www.ncbi.nlm.nih.gov/Traces/wgs/?val=GJZN01
